# mRNA Expression Profiles from Whole Blood Associated with Vasospasm in Patients with Subarachnoid Hemorrhage

**DOI:** 10.1007/s12028-019-00861-x

**Published:** 2019-10-08

**Authors:** Huichun Xu, Boryana Stamova, Bradley P. Ander, Ben Waldau, Glen C. Jickling, Frank R. Sharp, Nerissa U. Ko

**Affiliations:** 1 Department of Medicine, University of Maryland, College Park, USA; 2 Department of Neurology, University of California at Davis, 2805 50th St., Sacramento, CA 95817 USA; 3 Neurosurgery, University of California at Davis, Sacramento, USA; 4 Department of Neurology, University of Alberta, Edmonton, Canada; 5 Department of Neurology, University of California at San Francisco, San Francisco, USA

**Keywords:** RNA, Microarray, Vasospasm, Subarachnoid hemorrhage, Aneurysm, Delayed cerebral ischemia (DCI)

## Abstract

**Background:**

Though there are many biomarker studies of plasma and serum in patients with aneurysmal subarachnoid hemorrhage (SAH), few have examined blood cells that might contribute to vasospasm. In this study, we evaluated inflammatory and prothrombotic pathways by examining mRNA expression in whole blood of SAH patients with and without vasospasm.

**Methods:**

Adult SAH patients with vasospasm (*n* = 29) and without vasospasm (*n* = 21) were matched for sex, race/ethnicity, and aneurysm treatment method. Diagnosis of vasospasm was made by angiography. mRNA expression was measured by Affymetrix Human Exon 1.0 ST Arrays. SAH patients with vasospasm were compared to those without vasospasm by ANCOVA to identify differential gene, exon, and alternatively spliced transcript expression. Analyses were adjusted for age, batch, and time of blood draw after SAH.

**Results:**

At the gene level, there were 259 differentially expressed genes between SAH patients with vasospasm compared to patients without (false discovery rate < 0.05, |fold change| ≥ 1.2). At the exon level, 1210 exons representing 1093 genes were differentially regulated between the two groups (*P* < 0.005, ≥ 1.2 |fold change|). Principal components analysis segregated SAH patients with and without vasospasm. Signaling pathways for the 1093 vasospasm-related genes included adrenergic, P2Y, ET-1, NO, sildenafil, renin–angiotensin, thrombin, CCR3, CXCR4, MIF, fMLP, PKA, PKC, CRH, PPAR*α*/RXR*α*, and calcium. Genes predicted to be alternatively spliced included IL23A, RSU1, PAQR6, and TRIP6.

**Conclusions:**

This is the first study to demonstrate that mRNA expression in whole blood distinguishes SAH patients with vasospasm from those without vasospasm and supports a role of coagulation and immune systems in vasospasm.

**Electronic supplementary material:**

The online version of this article (10.1007/s12028-019-00861-x) contains supplementary material, which is available to authorized users.

## Introduction

Subarachnoid hemorrhage (SAH) morbidity and mortality is high and increases with Hunt–Hess grade [1]. Vasospasm occurs in approximately 70% of SAH patients, and delayed cerebral ischemia (DCI) occurs in approximately one quarter to one-third of patients; both are associated with worse outcomes [2]. Since vasospasm can occur without DCI, and preventing vasospasm does not prevent DCI, vasospasm combined with additional factors including pro-coagulant and pro-inflammatory/immune mechanisms has been postulated to contribute to DCI [3, 4].

Most previous SAH biomarker studies examined either cerebrospinal fluid (CSF), serum, or plasma [5, 6]. Molecules released from injured vessels, neurons, glia, and other cells can be released into plasma/serum. These can act on leukocytes, red blood cells, platelets, and other blood cells to contribute to the risk of vasospasm and possibly DCI. Though there are many studies of blood plasma/serum following SAH, there are few studies of the blood cells themselves in humans. Thus, in this study we examined the entire transcriptome using exon microarrays to assess RNA expression in whole blood which includes leukocytes, platelets, and other cells in SAH patients with vasospasm compared to SAH patients without vasospasm. Angiographic vasospasm was the primary end point in this study mainly because it was relevant when the cohort study was designed and initiated. In addition, there were too few DCI events in the final cohort to analyze in this pilot study.

## Methods

We performed a nested case control study of vasospasm in a prospective cohort study completed in 2012 of adult SAH patients admitted to University of California at San Francisco (UCSF). As part of the original cohort study, samples, including mRNA, were collected at the time of enrollment. The study complied with the principles of the Declaration of Helsinki (1964), and Institutional Review Board approval was obtained from UCSF and UC Davis. Informed consent was obtained from all individual participants or their legal surrogates included in this study. Patients with angiographic vasospasm (*n* = 29) and those without vasospasm (*n* = 21) were matched by sex, race/ethnicity, and aneurysm treatment with coil embolization. All patients had diagnostic testing with digital subtraction angiography (DSA). As part of standard clinical care, patients were monitored for vasospasm with transcranial Doppler (TCD) and serial clinical exams. Patients with elevated TCD velocities or clinical change without alternative explanation were assessed for possible vasospasm. Diagnosis of vasospasm was always confirmed by gold standard cerebral angiogram (DSA). Most patients had head computed tomography (CT), and fewer had brain magnetic resonance imaging as part of their routine clinical care. There was no pre-specified delayed imaging in the original cohort. No patient had clinical evidence of delayed cerebral ischemia (DCI) or infarct on CT at the time of blood draw, but patients without delayed imaging could have had clinically silent DCI. All patients had standard clinical management of SAH and vasospasm.

### Blood Collection and Processing

Whole blood (15 mL) was collected from each subject into six PAXgene tubes by venipuncture and stored frozen at − 80 °C after 2 h at room temperature. One blood draw was performed on each subject, with the time of blood draw being 10.9 ± 5.7 days in patients with vasospasm, and 8.5 ± 3.7 days in SAH patients without vasospasm (*P* = 0.059, Table 1). For the vasospasm patients, the blood draw was performed at the time vasospasm was first identified. Total RNA was isolated according to the manufacturer’s protocol (PAXgene blood RNA kit; Pre-AnalytiX) on an automated workstation QIAcube (Qiagen, Valencia, CA). Most RNA from a PAXgene tube is from polymorphonuclear cells (neutrophils, basophils, and eosinophils), mononuclear cells (lymphocytes and macrophages/monocytes), platelets and platelet precursors, and red blood cells and their precursors.

**Table 1:** Demographics of SAH patients with vasospasm and SAH patients without vasospasm

	Vasospasm	Non-vasospasm	*P* value
Number of subjects	29	21	
Sex (male/female)	7/22	6/15	0.738
Age (years)	53 ± 10	62 ± 17	0.025
Caucasian/non-Caucasian	23/6	17/4	1.000
Blood sample collection time:			
Days after onset of SAH	10.9 ± 5.7	8.5 ± 3.7	0.059
Fisher group			
1	0	1	0.858
2	8	5	
3	19	13	
4	2	2	
Hunt and Hess grade			
1	3	3	0.276
2	10	10	
3	9	5	
4	5	2	
5	2	1	
Treatment (coil/clip)	26/3	20/1	0.630

The numbers of patients in each Fisher group are indicated, and the numbers in each Hunt and Hess Grade are indicated. Most of the patients in both groups were treated by coiling

*SAH* subarachnoid hemorrhage

RNA quality and quantity were assessed using the Nanodrop ND-1000 (Nanodrop Inc., Wilmington, DE, USA) and Agilent 2100 Bioanalyzer (Agilent Technologies Inc., Foster City, CA, USA). RNA samples had an *A*_260_/*A*_280_ absorbance ratio greater than 2.0 and an RNA integrity number greater than 7.

### Exon microarray Processing

Twenty nanogram RNA samples were amplified using the WT-Ovation™ Pico RNA Amplification System (NuGEN, San Carlos, CA) with the exon module. The samples were fragmented and labeled using the FL-Ovation™ cDNA Biotin Module V2 (NuGEN, San Carlos, CA). Hybridization, washing, and laser scanning were performed according to the Affymetrix Human Exon 1.0 ST protocol (Affymetrix, Santa Clara, CA).

### Data Analysis

Raw data (Affymetrix.CEL files) were imported into Partek Genomics Suite 6.4 (Partek Inc., St. Louis, MO). Core probesets which are supported by the most reliable evidence from RefSeq and full-length mRNA GenBank records containing complete coding region of a gene information were included for the analysis. Genomic annotation was based on the March 2006 human reference sequence (NCBI Build 36.1). Probeset normalizations were performed using Robust Microarray Analysis with log_2_ transformation.

For gene-level analysis, expression from all probesets for the exons in a gene was averaged to produce a single value for each gene. To assess genes or exons differentially regulated between SAH patients with vasospasm and those without vasospasm, an analysis of covariance (ANCOVA) was conducted and included sample batch, patient age, and days after SAH onset as covariates. Patient age and days after SAH were included as covariates since they were significantly (*P* < 0.05) different, or nearly so (*P* = 0.054), between vasospasm and no vasospasm groups (Table 1). Alternative splicing was assessed using Partek alternative splicing analysis of variance. Demographic data were analyzed with Student’s *t* test or Fisher’s exact tests. The distribution of Hunt and Hess grade and Fisher group were compared between cases and controls using the ptrend test.

Genes that were differentially regulated at either the whole gene or exon level between subjects with and without vasospasm were further assessed using Ingenuity Pathways Analysis (IPA 8.0, Ingenuity^®^ Systems). This explored molecular functions, interactions, and signaling pathways associated with the identified genes. Fischer’s exact test was used to calculate a *P* value describing the probability that a given biological function/pathway was assigned to that data set due to chance alone.

## Results

### Demographics

The SAH patients with and without vasospasm were matched based on group and sex. There were no significant differences in race, sex, Fisher group, Hunt and Hess grade, and aneurysm treatment methods between SAH patients with and without vasospasm (Table 1). SAH patients with vasospasm were younger than those without vasospasm (53 ± 10 vs. 62 ± 17 years, *P* = 0.025), like other SAH cohorts. Time to blood sample collection in patients with vasospasm (10.9 ± 5.7 days after SAH onset) was slightly longer compared to those without vasospasm (8.5 ± 3.7 days after SAH onset, *P* = 0.059). Thus, age and time of blood draw after SAH were included as covariates in the ANCOVA model.

### Differential Expression at the Gene Level

For the gene-level analysis, the expression value of each exon for a given gene was averaged to yield a single expression value for each gene. With a false discovery rate of ≤ 0.05 to account for multiple comparisons, and a ≥ 1.2-|fold change| (to help ensure biological significance), there were 276 probes representing 259 genes that were differentially regulated between SAH patients with vasospasm and those without vasospasm. A complete list of the genes is shown in Supplementary Table 1. The false discovery rate was based upon the numbers of expressed genes, where an expressed gene was defined as above background levels in all patient samples.

### Differential Expression Pattern at Exon Level

To determine whether SAH patients with vasospasm have different gene expression profiles from those patients without vasospasm at the exon level, the ANCOVA analysis model was applied to exon level data in which each exon was individually assessed. At *P* ≤ 0.005 and ≥ 1.2-|fold change| criteria, there were 1210 exons from 1093 unique genes identified as differentially regulated between SAH patients with vasospasm and those without vasospasm (Supplementary Table 2).

**Fig. 1:**
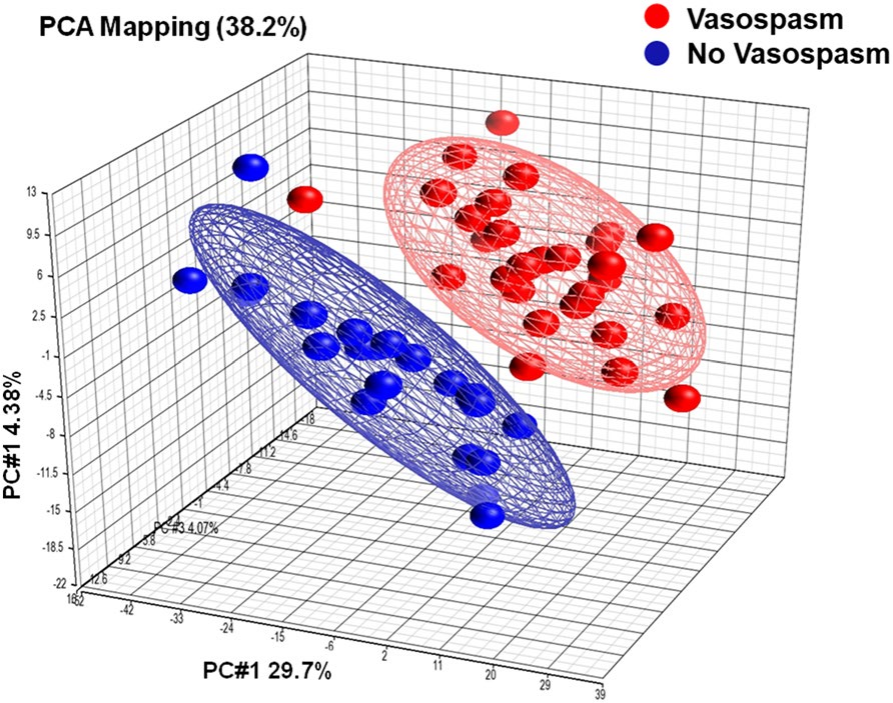
Principal components analysis (PCA). The 1210 exons that were differentially expressed in patients with vasospasm versus those without vasospasm (*P* < 0.005 and |fold change| > 1.2) were used for a PCA. The top three principal components were represented on the *X*, *Y*, and *Z* axes. The three components account for 72.3% of the total estimated variance. Each sphere represents one subject, red being SAH patients with vasospasm and blue being SAH patients without vasospasm. The distance between spheres in the 3-D space shows their differences based on the expression pattern. Each of the two ellipsoids represents a two-standard deviation space from the mean of each group of samples

A principal component analysis (PCA) was performed based on expression of the 1210 exons. PCA mapping projects the expression pattern of an individual into a multidimensional space for visual inspection of their similarities and dissimilarities. The PCA results showed a clear segregation of the exon expression patterns between SAH patients with vasospasm and without (Fig. 1).

Function annotation analysis of the identified 1093 genes represented by 1210 exons was performed in the Ingenuity database. The major signaling pathways associated with these transcripts included cardiovascular signaling, neurological signaling, immune response signaling, stress response signaling, and other intracellular signaling (Table 2). Of interest, several gene products in nitric oxide, vascular endothelial growth factor (VEGF) and calcium signaling had differential expression at the exon level between patients with vasospasm and those without vasospasm including VEGF receptors, PI3 K, and PKC in the VEGF/NO pathway, and L-type calcium channel, PDE2, IP3R, RyR2, and SERCA (Supplementary Table 2).

**Table 2:** Significant pathways for the 1093 genes differentially expressed in SAH patients with vasospasm compared to those without vasospasm

Classification	Canonical pathways	*P* value	#
Cardiovascular signaling	Cardiac β-adrenergic signaling	0.0026	17
	α-Adrenergic signaling	0.0081	12
	P2Y Purigenic receptor signaling pathway	0.0148	14
	Endothelin-1 signaling	0.0178	18
	Nitric oxide signaling in the cardiovascular system	0.0240	10
	Cellular effects of sildenafil (Viagra)	0.0282	14
	Renin–angiotensin signaling	0.0339	12
	Thrombin signaling	0.0457	18
Neurological signaling	Synaptic long-term depression	0.0008	19
	Synaptic long-term potentiation	0.0055	14
	GNRH signaling	0.0093	15
	Axonal guidance signaling	0.0107	34
	CREB signaling in neurons	0.0120	19
	Neuropathic pain signaling in dorsal horn neurons	0.0200	12
Inflammation response signaling	CCR3 signaling in eosinophils	0.0102	14
	CXCR4 signaling	0.0288	16
	MIF mediated glucocorticoid regulation	0.0363	5
	fMLP signaling in neutrophils	0.0380	12
Intracellular signaling	Protein kinase A signaling	0.0178	28
	Phospholipase C signaling	0.0191	22
	Calcium signaling	0.0309	17
Stress response signaling	PPAR*α*/RXR*α* activation	0.0182	17
	Corticotropin-releasing hormone signaling	0.0363	12
Cell cycle signaling	Molecular mechanisms of cancer	0.0055	33
	Breast cancer regulation by Stathmin1	0.0083	21

*CCR3* CC chemokine receptor-3, *CREB* cAMP responsive element binding protein, *CXCR4* C-X-C motif chemokine receptor-4, *fMLP* N-formyl-Met-Leu-Phe, *GNRH* gonadotropin-releasing hormone, *MIF* Migration inhibitory factor, *PPARα/RXRα* The peroxisome proliferator-activated receptor *α*/retinoid X receptor *α*, *SAH* subarachnoid hemorrhage

### Alternative Splicing Associated with Vasospasm

Finally, an alternative splicing ANCOVA was performed in Partek with sample batch, patient age, and time after SAH as covariates. To identify the most likely spliced transcripts, additional selection criteria included: (1) exons with a log (base 2)-transformed expression value < 3.0 in all samples and no significant expression difference between the two groups at *P* < 0.05 were excluded; (2) transcripts which exhibited differential expression between the two groups (criteria: *P* < 0.05) were excluded; and (3) transcripts with more than 25 probesets were excluded to simplify the analysis. With *P* ≤ 0.05, 719 genes were predicted to be alternatively spliced between SAH patients with and without vasospasm. Of these, four passed a false discovery rate of < 5% false positives: interleukin 23 alpha subunit p19 (IL23A); Ras suppressor protein 1(RSU1); progestin and adipoQ receptor family member VI (PAQR6); and thyroid hormone receptor interactor 6 (TRIP6). For example, the 3rd exon of IL23A in vasospasm patients exhibited 1.68-fold higher expression than non-vasospasm patients, while whole gene expression for IL23A did not differ between groups.

## Discussion

This is the first genome-wide study of mRNA expressed in whole blood of patients with aneurysmal SAH who developed vasospasm as compared to those who did not. There were 259 genes, 1210 exons from 1093 genes, and 719 alternatively spliced transcripts differentially expressed for SAH patients with vasospasm compared to those without vasospasm. The signaling pathways over-represented by the 1093 genes included stress signaling, immune response signaling, cardiovascular signaling, calcium signaling, and cell growth/death signaling. These results suggest that changes in peripheral blood clotting, immune and inflammatory pathways likely impact cerebral blood vessels following SAH and contribute to the occurrence of vasospasm. Some of these might contribute to DCI, though DCI was not studied here and needs to be evaluated in the future.

The specificity of the pathways associated with SAH-related vasospasm (SAH-V) can be appreciated by comparing the results of this study to our recent whole-genome study of blood in patients with intracerebral hemorrhage (ICH) compared to controls [7]. Of the 1213 genes expressed in ICH [8] and the 247 expressed in SAH-V (current study), only 12 overlapped (*P* > 0.2). Of the 378 alternatively spliced transcripts in ICH [7] and the 719 in SAH-V, only 16 overlapped (*P* > 0.2). Of the 65 pathways identified for ICH [7] and the 25 for SAH-V (current study), five overlapped including alpha adrenergic signaling, renin–angiotensin signaling, phospholipase C, corticotropin-releasing hormone signaling, and cancer mechanisms. However, the number of shared pathways was not statistically significant (*P* = 0.11). Thus, the blood immune and inflammatory responses that follow ICH are different from those associated with vasospasm that results from SAH.

A previous whole-genome study of blood following SAH identified 135 differentially expressed genes in blood compared to controls, with a subgroup of 16 genes that distinguished SAH from controls [9]. In that study, bloods were taken at various times after SAH, which showed activation of genes in neutrophils and monocytes, with suppression of genes in lymphocytes [9]. Though vasospasm was not studied, approximately 15% of the genes/pathways overlapped from our study and theirs [9] and few genes in either study overlap those in the walls of ruptured aneurysms [10].

Pathways enriched with the genes associated with vasospasm in our study included alpha and beta adrenergic signaling. This is consistent with previous findings that plasma and CSF levels of epinephrine are elevated in SAH, and that epinephrine independently predicts morbidity and mortality in SAH [5]. Systemic epinephrine/norepinephrine contributes to cardiac abnormalities in SAH [11], and there is loss of peri-vascular adrenergic fibers following SAH [12]. P2Y purinergic receptor signaling was also associated with vasospasm. In vitro studies suggest purinergic signaling triggers astrocyte end foot high-amplitude calcium signaling and causes inversion of neurovascular coupling after SAH [13]. P2 receptor inhibition using suramin restored vasodilatory neurovascular coupling after SAH [13].

The results also implicate endothelin-1 and nitric oxide signaling in vasospasm related to SAH. These are not surprising given one is a potent vasoconstrictor and the other a potent vasodilator, respectively. ET-1 is increased in CSF and blood of SAH patients [5]. ET-1 causes vasospasm, and ET-1 antagonists can reverse vasospasm in experimental models and in patients, but do not improve outcomes in SAH patients [14]. Hemoglobin is a nitric oxide scavenger and thus could directly lead to vasoconstriction. However, vascular smooth muscle cells become unresponsive to NO during SAH [15], perhaps helping to explain why targeting NO in SAH may not be very efficacious [16].

Our data also implicate vascular sildenafil (Viagra) signaling. Systemic sildenafil in patients with SAH decreases systemic blood pressure without affecting cerebral perfusion pressure or cerebral blood flow [17]. However, the drug did appear to decrease cerebral vasospasm [18].

The renin–angiotensin pathway was also associated with vasospasm. Loss of body sodium can herald the onset or worsening of clinical vasospasm as the renin–angiotensin–aldosterone system is activated in a delayed manner [19]. Renin–angiotensin genes (CARAS study) are being examined for possible association with SAH [20].

Thrombin signaling was also associated with vasospasm following SAH in our study. SAH produces large amounts of thrombin, which has little contractile effect on normal brain arteries. However, following SAH thrombin enhances and prolongs vascular contraction because of the upregulation of its PAR(1) receptor and impairment of receptor desensitization in arterial smooth muscle [21]. Since receptor desensitization is impaired after SAH, thrombin-induced contraction persists even after thrombin stimulation ceases. An intrathecal PAR(1) antagonist prevents the PAR(1) upregulation and the increased reactivity to thrombin and helps resolve vasospasm [21]. Not only would thrombin promote vasospasm, but it would also promote clotting and thus contribute to DCI [22]. Thrombin activation of the TGF-beta pathway may also contribute to communicating hydrocephalus following SAH [23].

Inflammatory response signaling for CCR3, CSCR4, MIF, and fMLP was also prominent in our vasospasm data. MIF is an independent predictor of 6 month outcome in SAH patients [24]. MIF enhances blood brain barrier permeability [25] and worsens stroke in some models [26], though it appears to protect against stroke in other studies [27]. MIF binds to the CXCR4 receptor [28]. A CXCR4 antagonist reverses the neurogenesis and behavioral recovery produced by forced limb use following rodent stroke [29]. Physical exercise regulates neural stem cells via CXCR4 in rats after stroke [30]. CXCR4 also regulates vascular α-adrenergic function [31].

fMLP signaling in neutrophils could have profound effects on clotting/thrombosis resulting in DCI following SAH. fMLP stimulates tissue factor in neutrophils which promotes clotting [32]. Neutrophil Extracellular Traps are composed of fibers released from neutrophils that capture platelets, increase fibrin deposition, and promote clotting [33, 34]. Our data also suggest a role for CCR3 signaling in eosinophils in vasospasm, a cell type not previously implicated in SAH.

Calcium signaling is implicated in SAH/vasospasm since nimodipine is the only Food and Drug Administration approved drug to decrease the incidence of DCI [35]. PKA signaling regulates NO [36]. PKC regulates ET signaling [37]. PPAR alpha promotes formation and rupture of aneurysms in mice [38], but also decreases vasospasm-related cytokines [39] and hemoglobin-induced TLR4 expression in vascular myocytes [40].

The roles of the neurological signaling pathways are unclear, since there are no neurons in blood. Thus, these pathways may be performing similar functions in leukocytes. For example, nimodipine acts on TrkB receptors, found on some leukocytes, to increase levels of phosphorylated Akt and CREB which may modify immune cell function as well as that of neurons [41, 42]. Mild hypothermia protects against early brain injury in rats following subarachnoid hemorrhage via the TrkB/ERK/CREB signaling pathway [42]. A cannabinoid receptor type 2 agonist attenuates apoptosis by activation of phosphorylated CREB-Bcl2 pathway after SAH in rats [43].

Synaptic long-term depression and synaptic long-term potentiation were among the significant pathways associated with vasospasm. This could indicate alteration of these pathways in hippocampus and could contribute to the memory problems seen in SAH patients particularly those with vasospasm [44, 45]. Depletion of neutrophils decreased inflammation and vasospasm, preserved Long Term potentiation and N-methyl-d-aspartate function, and improved memory function in experimental SAH [46].

As this is a first pilot feasibility study of mRNA expression in SAH, there are limitations in sample size, timing of sample collection, and outcomes measured. In the original cohort, timing of blood samples was not pre-specified. We restricted subjects to the earliest blood samples collected, but there was still variability in timing, and limited early timepoints for analysis. Vasospasm was the primary outcome studied here because it was still an important end point when the cohort study was designed and initiated. Furthermore, there were too few DCI events (*n* = 5/29) to analyze separately. Imaging outcomes of DCI were not routinely acquired in the cohort, and we may have missed clinically silent events. Missing silent vasospasm and DCI could cause misclassification bias. Despite these limitations, we were able to demonstrate promising results and proof of principle that will better inform future studies. Therefore, future studies will need to compare SAH patients using modern definitions of DCI to identify genes and pathways associated with these outcomes. Larger sample sizes will allow for better matching of patients and adjustment for clinical confounders. Timing of samples, including multiple timepoints with pre-specified outcome measures, will be important. A replication study to account for multiple comparisons needs to be performed to validate results.

## Conclusions

These studies lay the foundation for future studies of genes and pathways associated with DCI, in SAH patients with or without vasospasm.

## Electronic supplementary material

Below is the link to the electronic supplementary material.

Supplementary material 1 (PDF 127 kb)

Supplementary material 2 (PDF 344 kb)
