# Supplementary material for: mRNA Expression Profiles from Whole Blood Associated with Vasospasm in Patients with Subarachnoid Hemorrhage
Source: Neurocrit Care. 2019 Oct 8;33(1):82–9. doi: 10.1007/s12028-019-00861-x (PMC7392923; doi:10.1007/s12028-019-00861-x)
Supplement: Supplementary file 1 — Supplementary material 1 (PDF 127 kb) [file 12028_2019_861_MOESM1_ESM.pdf]

Gene Symbol	Transcript Cluster ID	p-value (Vasospasm vs. No vasospasm)
ZMAT4	3132616	9.69E-05
OR2D3	3318982	4.07E-04
MGC39372	2939014	5.78E-04
RGS18	2372719	9.26E-04
ALDH3B2	3379091	1.41E-03
MAGOHB	3444195	1.73E-03
ST6GALNAC3	2342738	1.87E-03
ZNF519	3800165	1.90E-03
ZNF493	3826601	1.97E-03
NCRNA00164	2576788	2.18E-03
PPA2	2780522	2.38E-03
ZNF417	3872521	2.48E-03
LOC84740	2717481	3.28E-03
KLK1	3868681	3.39E-03
CHN1	2587961	3.60E-03
	3511158	3.62E-03
RBM12B	3144760	3.63E-03
OR10S1	3395811	3.66E-03
ELMOD2	2745067	3.79E-03
CLK1	2594497	3.81E-03
ALOX12	3708160	4.01E-03
HOXB1	3761274	4.10E-03
TLR10	2766192	4.19E-03
APOB48R	3686635	4.21E-03
FLJ39739	2433605	4.33E-03
ZNF107	3004628	4.36E-03
ZNF611	3869714	4.37E-03
PSG9	3864286	4.60E-03
OR10K2	2439314	4.63E-03
LOC728212	3937967	5.00E-03
ATL2	2548776	5.13E-03
ODF2L	2421121	6.03E-03
IFNA5	3201255	6.09E-03
C21orf66	3929395	6.18E-03
ZNF419	3843275	6.25E-03
ACTA2	3299504	6.29E-03
ZMAT1	4016001	6.53E-03
	3335640	6.66E-03
OR52M1	3318141	8.08E-03
ZNF260	3860491	8.62E-03
ZNF20	3851250	8.72E-03
ARL17P1	3760268	8.80E-03
CBR4	2793054	8.88E-03
ZNF141	2713950	8.98E-03
	3430125	9.26E-03
CAPZA3	3407016	9.29E-03

KATNA1	2978957	9.44E-03
OR6V1	3028923	9.47E-03
TAAR6	2926147	9.70E-03
HOOK1	2338625	9.70E-03
POLG2	3766861	9.81E-03
SPINK5L2	2834574	1.01E-02
ATP6V0A1	3721718	1.02E-02
ARHGAP12	3283920	1.03E-02
PHOSPHO2	2514497	1.04E-02
TAF7	2878622	1.04E-02
ZBED5	3362934	1.08E-02
ZNF226	3835494	1.08E-02
LRRC6	3154136	1.10E-02
ZNF180	3864921	1.11E-02
PLEKHF2	3107828	1.13E-02
KRR1	3462693	1.16E-02
	2763154	1.18E-02
PIBF1	3493448	1.20E-02
CCRL2	4047185	1.22E-02
ERV3	3053380	1.23E-02
PMS1	2519981	1.27E-02
MUT	2956438	1.27E-02
ZNF585A	3860737	1.30E-02
LOC133874	2840238	1.32E-02
WDR75	2519756	1.34E-02
H2BFXP	3985774	1.35E-02
ABAT	3647421	1.37E-02
FTHL3P	2545841	1.40E-02
FAM122C	3991814	1.45E-02
	3335793	1.45E-02
ZFAND5	3209623	1.48E-02
OR13J1	3204987	1.49E-02
LOC84931	2573326	1.53E-02
GORAB	2366753	1.58E-02
SENP7	2686646	1.59E-02
TSGA10	2566586	1.61E-02
GOLGA8B	3617574	1.62E-02
SLC35A5	2636185	1.62E-02
AKR1A1	2334374	1.62E-02
MGAT4A	2566414	1.64E-02
SH3YL1	2537109	1.65E-02
SNRPN	3584728	1.67E-02
RABGGTB	2342624	1.68E-02
UTP15	2815455	1.68E-02
ZNF701	3840372	1.70E-02
TIGD1	2603897	1.71E-02
LOC400713	3840194	1.72E-02

POLI	3788833	1.75E-02
C3orf63	2677653	1.76E-02
FASTKD3	2847710	1.77E-02
BHLHA15	3014227	1.78E-02
ERO1LB	2462329	1.78E-02
SCFD1	3531032	1.80E-02
PSMA3	3537747	1.81E-02
PIGH	3569339	1.83E-02
STOX1	3249978	1.83E-02
BLOC1S2	3303392	1.84E-02
LYRM7	2828135	1.85E-02
MRPL13	3150797	1.88E-02
DRAM2	2427720	1.92E-02
NAT5	3878934	1.94E-02
CCDC53	3468225	1.95E-02
MPO	3764245	1.95E-02
ANKRA2	2862380	1.96E-02
TRNT1	2608156	1.99E-02
MTFR1	3101385	2.01E-02
BMS1	3938817	2.02E-02
	2412222	2.03E-02
	2333667	2.04E-02
HIST1H3F	2946364	2.07E-02
EBPL	3513856	2.07E-02
MAD2L1	2783715	2.07E-02
UACA	3631397	2.08E-02
ZNF267	3657367	2.10E-02
XKR6	3124333	2.12E-02
ZNF654	2632036	2.14E-02
CWF19L2	3389745	2.15E-02
FLJ30679	3672640	2.15E-02
KRTAP4-12	3756750	2.15E-02
TTC14	2654306	2.18E-02
C1GALT1C1	4019967	2.25E-02
ZNF83	3869650	2.27E-02
MFN1	2653932	2.27E-02
	3831272	2.28E-02
	2969796	2.29E-02
ESCO1	3800779	2.31E-02
CCDC126	2992963	2.33E-02
HOXD12	2516793	2.34E-02
C4orf27	2793310	2.36E-02
ZNF26	3439063	2.38E-02
LYPLAL1	2380785	2.41E-02
ANKRD32	2820622	2.44E-02
HACL1	2664395	2.47E-02
THAP6	2731757	2.47E-02

HEATR1	2462511	2.47E-02
PRPF39	3534201	2.49E-02
ESF1	3898224	2.50E-02
BNIP3	3314040	2.53E-02
	3958267	2.54E-02
METTL10	3311342	2.54E-02
AHI1	2975385	2.54E-02
RPL5	2346863	2.57E-02
LY96	3103523	2.63E-02
CENPQ	2909723	2.65E-02
C1orf103	2427688	2.67E-02
NCRNA00201	2464484	2.69E-02
ZNF718	2713837	2.70E-02
ZNF841	3869396	2.71E-02
NEK1	2793221	2.71E-02
TMEM161B	2866045	2.72E-02
TMEM168	3068476	2.73E-02
ZNF514	2564634	2.75E-02
TOMM7	3041260	2.75E-02
ZNF528	3840224	2.75E-02
PACS2	3554622	2.81E-02
GSTM1	2350981	2.81E-02
PPA1	3293280	2.85E-02
CALML6	2316218	2.85E-02
	2446358	2.88E-02
NCBP2	2713074	2.90E-02
ELP2	3784727	2.92E-02
KRTAP2-4	3756723	2.92E-02
PUS3	3396736	2.93E-02
TTC30B	2588965	2.93E-02
NOC3L	3301011	2.96E-02
SPRR2A	2435858	2.99E-02
GSTM2	2350952	3.00E-02
ZMYM5	3504054	3.04E-02
CCDC109B	2739160	3.05E-02
IFT80	2703133	3.05E-02
ZNF431	3826504	3.06E-02
ZNF277	3019401	3.07E-02
DCUN1D5	3388914	3.08E-02
SH2D1A	3989826	3.10E-02
FLJ38379	2536965	3.11E-02
EFHA1	3504791	3.11E-02
INADL	2339139	3.12E-02
ZNF253	3826041	3.13E-02
NIPSNAP3A	3182957	3.16E-02
OR10A4	3318976	3.19E-02
GIN1	2869275	3.21E-02

CEP135	2727976	3.22E-02
TCF7L2	3264621	3.23E-02
PRR23C	2697721	3.29E-02
ZNF273	3004768	3.31E-02
SRFBP1	2826064	3.32E-02
ZNF721	2756404	3.37E-02
ACADSB	3268588	3.37E-02
HSPB11	2413519	3.38E-02
SKAP1	3761164	3.41E-02
IGSF9B	3399456	3.43E-02
EIF4H	3008144	3.44E-02
LYRM5	3408573	3.45E-02
MRPS33	3076489	3.45E-02
LUC7L3	3726772	3.49E-02
	2437554	3.52E-02
XRCC4	2818454	3.52E-02
TRMT61B	2546285	3.53E-02
	3874584	3.53E-02
NPBWR1	3098213	3.53E-02
ANAPC10	2788143	3.54E-02
PPP1R2	2712147	3.55E-02
LSM8	3020804	3.56E-02
C19orf59	3819088	3.58E-02
GPR171	2701018	3.61E-02
MRPL42	3426215	3.62E-02
INPP4B	2787459	3.63E-02
	2464129	3.64E-02
MRPS31	3510925	3.64E-02
ATF1	3414695	3.64E-02
RPAP2	2346738	3.66E-02
ORMDL1	2591942	3.69E-02
C7orf23	3060051	3.69E-02
NBPF22P	2818884	3.74E-02
FKSG83	2899874	3.75E-02
NARG2	3627363	3.78E-02
PHF10	2986084	3.81E-02
PYGO1	3625440	3.85E-02
MYNN	2651671	3.86E-02
ZNF461	3860596	3.87E-02
ERGIC2	3448975	3.90E-02
	2869558	3.90E-02
KRTAP26-1	3928477	3.92E-02
TXNDC9	2566740	3.93E-02
CBR3	3919834	3.93E-02
ARHGAP15	2508611	3.96E-02
USP1	2339414	3.98E-02
GKAP1	3212189	4.02E-02

REEP3	3249043	4.05E-02
TAS2R1	2848118	4.09E-02
TTC37	2867693	4.10E-02
KIAA1586	2911257	4.14E-02
ACADM	2342576	4.16E-02
	3184925	4.22E-02
CCDC23	2409069	4.23E-02
C8orf38	3107724	4.25E-02
PDCD10	2704188	4.27E-02
KPNA5	2922840	4.28E-02
NUDCD1	3148796	4.29E-02
LPAR6	3513514	4.30E-02
	2901698	4.31E-02
CCDC82	3387771	4.33E-02
EEF1E1	2940920	4.33E-02
TMEM14A	2910364	4.34E-02
KRTAP19-1	3928522	4.38E-02
C7orf36	2998333	4.40E-02
STX3	3332131	4.40E-02
ITGB3BP	2416218	4.43E-02
KIAA1147	3076753	4.45E-02
CSTA	2638869	4.49E-02
SMARCAD1	2736259	4.52E-02
ATP6V1C1	3110171	4.54E-02
TRAT1	2635349	4.55E-02
SLC25A36	2645275	4.64E-02
NMD3	2650538	4.67E-02
ZNHIT3	3719112	4.71E-02
PPIG	2514441	4.72E-02
OR4C12	3373046	4.74E-02
NEK2	2454444	4.80E-02
PIGK	2418929	4.81E-02
NAE1	3695268	4.82E-02
CSNK1G3	2826550	4.82E-02
LAIR2	3841506	4.84E-02
ATP5L	3351359	4.84E-02
USPL1	3484005	4.86E-02
UBA3	2681195	4.86E-02
TTC39B	3199662	4.89E-02
ZNF644	2422517	4.93E-02
COX16	3570454	4.95E-02
ZNF627	3821301	4.97E-02

Fold-Change (Vasospasm vs. No vasospasm)
-1.22
-1.37
1.32
1.32
-1.24
1.37
1.25
1.29
1.32
-1.40
1.20
1.42
-1.26
-1.22
1.23
1.56
1.26
-1.21
1.36
1.37
-1.27
-1.23
1.34
-1.44
1.38
1.36
1.23
-1.24
-1.20
-1.45
1.21
1.29
-1.28
1.43
1.25
-1.22
1.38
1.66
-1.26
1.24
1.24
1.58
1.31
1.43
1.28
-1.24

1.20
-1.22
1.44
1.25
1.25
-1.21
-1.36
1.20
1.38
1.32
1.39
1.29
1.62
1.29
1.38
1.23
-1.21
1.35
-1.27
1.51
1.26
1.23
1.20
1.28
1.31
1.28
-1.23
1.22
1.22
-1.35
1.21
-1.33
-1.22
1.23
1.21
1.27
1.49
1.22
-1.23
1.25
1.27
1.56
1.31
1.24
1.25
1.48
1.28

1.22
1.32
1.30
-1.22
1.21
1.27
1.20
1.51
-1.20
1.22
1.49
1.21
1.22
1.21
1.27
-1.26
1.26
1.22
1.23
1.30
1.28
-1.47
-1.20
-1.22
1.40
1.27
1.27
1.26
1.24
1.22
-1.20
-1.42
1.29
1.21
1.30
1.22
1.35
1.22
1.21
1.23
-1.22
1.33
1.21
1.27
1.27
1.20
1.25

1.23
1.21
1.28
1.45
1.33
1.20
1.20
1.23
1.49
1.21
1.25
1.22
1.25
1.27
1.20
1.27
1.22
1.24
1.25
1.27
-1.21
-1.41
1.25
-1.21
-1.21
1.22
1.20
-1.23
1.22
1.22
1.28
-1.25
-1.32
1.22
1.34
1.22
1.23
1.24
1.20
1.23
1.25
1.21
1.22
1.28
1.32
-1.28
1.27

1.24
-1.22
-1.31
1.33
1.27
1.32
1.23
1.39
1.25
-1.24
1.28
1.31
1.42
1.20
1.26
1.33
1.25
1.22
1.23
1.39
1.29
1.40
-1.22
1.28
1.22
1.24
1.29
1.21
-1.23
1.22
1.21
1.21
-1.30
-1.22
1.22
1.30
-1.24
1.27
1.23
1.23
-1.22
-1.21
1.25
1.30
1.20
1.24
1.30

1.32
1.22
1.21
1.30
1.28
1.24
-1.35
1.21
1.35
1.28
1.25
1.36
-1.32
1.27
1.33
1.21
-1.25
1.29
-1.27
1.32
1.41
1.30
1.24
1.20
1.34
1.24
1.25
1.20
1.21
-1.22
-1.22
1.29
1.24
1.24
-1.71
1.33
1.22
1.22
1.21
1.23
1.35
1.20

Fold-Change (Vasospasm vs. No vasospasm) (Description)
Vasospasm down vs No vasospasm
Vasospasm down vs No vasospasm
Vasospasm up vs No vasospasm
Vasospasm up vs No vasospasm
Vasospasm down vs No vasospasm
Vasospasm up vs No vasospasm
Vasospasm up vs No vasospasm
Vasospasm up vs No vasospasm
Vasospasm up vs No vasospasm
Vasospasm down vs No vasospasm
Vasospasm up vs No vasospasm
Vasospasm up vs No vasospasm
Vasospasm down vs No vasospasm
Vasospasm down vs No vasospasm
Vasospasm up vs No vasospasm
Vasospasm up vs No vasospasm
Vasospasm up vs No vasospasm
Vasospasm down vs No vasospasm
Vasospasm up vs No vasospasm
Vasospasm up vs No vasospasm
Vasospasm down vs No vasospasm
Vasospasm down vs No vasospasm
Vasospasm up vs No vasospasm
Vasospasm down vs No vasospasm
Vasospasm up vs No vasospasm
Vasospasm up vs No vasospasm
Vasospasm up vs No vasospasm
Vasospasm down vs No vasospasm
Vasospasm down vs No vasospasm
Vasospasm down vs No vasospasm
Vasospasm up vs No vasospasm
Vasospasm up vs No vasospasm
Vasospasm down vs No vasospasm
Vasospasm up vs No vasospasm
Vasospasm up vs No vasospasm
Vasospasm down vs No vasospasm
Vasospasm up vs No vasospasm
Vasospasm up vs No vasospasm
Vasospasm down vs No vasospasm
Vasospasm up vs No vasospasm
Vasospasm up vs No vasospasm
Vasospasm up vs No vasospasm
Vasospasm up vs No vasospasm
Vasospasm up vs No vasospasm
Vasospasm up vs No vasospasm
Vasospasm down vs No vasospasm

Vasospasm up vs No vasospasm
Vasospasm down vs No vasospasm
Vasospasm up vs No vasospasm
Vasospasm up vs No vasospasm
Vasospasm up vs No vasospasm
Vasospasm down vs No vasospasm
Vasospasm down vs No vasospasm
Vasospasm up vs No vasospasm
Vasospasm up vs No vasospasm
Vasospasm up vs No vasospasm
Vasospasm up vs No vasospasm
Vasospasm up vs No vasospasm
Vasospasm up vs No vasospasm
Vasospasm up vs No vasospasm
Vasospasm up vs No vasospasm
Vasospasm up vs No vasospasm
Vasospasm down vs No vasospasm
Vasospasm up vs No vasospasm
Vasospasm down vs No vasospasm
Vasospasm up vs No vasospasm
Vasospasm up vs No vasospasm
Vasospasm up vs No vasospasm
Vasospasm up vs No vasospasm
Vasospasm up vs No vasospasm
Vasospasm up vs No vasospasm
Vasospasm up vs No vasospasm
Vasospasm down vs No vasospasm
Vasospasm up vs No vasospasm
Vasospasm up vs No vasospasm
Vasospasm down vs No vasospasm
Vasospasm up vs No vasospasm
Vasospasm down vs No vasospasm
Vasospasm down vs No vasospasm
Vasospasm up vs No vasospasm
Vasospasm up vs No vasospasm
Vasospasm up vs No vasospasm
Vasospasm up vs No vasospasm
Vasospasm up vs No vasospasm
Vasospasm down vs No vasospasm
Vasospasm up vs No vasospasm
Vasospasm up vs No vasospasm
Vasospasm up vs No vasospasm
Vasospasm up vs No vasospasm
Vasospasm up vs No vasospasm
Vasospasm up vs No vasospasm
Vasospasm up vs No vasospasm
Vasospasm up vs No vasospasm

Vasospasm up vs No vasospasm
Vasospasm up vs No vasospasm
Vasospasm up vs No vasospasm
Vasospasm down vs No vasospasm
Vasospasm up vs No vasospasm
Vasospasm up vs No vasospasm
Vasospasm up vs No vasospasm
Vasospasm up vs No vasospasm
Vasospasm down vs No vasospasm
Vasospasm up vs No vasospasm
Vasospasm up vs No vasospasm
Vasospasm up vs No vasospasm
Vasospasm up vs No vasospasm
Vasospasm up vs No vasospasm
Vasospasm up vs No vasospasm
Vasospasm down vs No vasospasm
Vasospasm up vs No vasospasm
Vasospasm up vs No vasospasm
Vasospasm up vs No vasospasm
Vasospasm up vs No vasospasm
Vasospasm up vs No vasospasm
Vasospasm down vs No vasospasm
Vasospasm down vs No vasospasm
Vasospasm down vs No vasospasm
Vasospasm up vs No vasospasm
Vasospasm up vs No vasospasm
Vasospasm up vs No vasospasm
Vasospasm up vs No vasospasm
Vasospasm up vs No vasospasm
Vasospasm up vs No vasospasm
Vasospasm down vs No vasospasm
Vasospasm down vs No vasospasm
Vasospasm up vs No vasospasm
Vasospasm up vs No vasospasm
Vasospasm up vs No vasospasm
Vasospasm up vs No vasospasm
Vasospasm up vs No vasospasm
Vasospasm up vs No vasospasm
Vasospasm up vs No vasospasm
Vasospasm up vs No vasospasm
Vasospasm down vs No vasospasm
Vasospasm up vs No vasospasm
Vasospasm up vs No vasospasm
Vasospasm up vs No vasospasm
Vasospasm up vs No vasospasm
Vasospasm up vs No vasospasm
Vasospasm up vs No vasospasm

Vasospasm up vs No vasospasm
Vasospasm up vs No vasospasm
Vasospasm up vs No vasospasm
Vasospasm up vs No vasospasm
Vasospasm up vs No vasospasm
Vasospasm up vs No vasospasm
Vasospasm up vs No vasospasm
Vasospasm up vs No vasospasm
Vasospasm up vs No vasospasm
Vasospasm up vs No vasospasm
Vasospasm up vs No vasospasm
Vasospasm up vs No vasospasm
Vasospasm up vs No vasospasm
Vasospasm up vs No vasospasm
Vasospasm up vs No vasospasm
Vasospasm up vs No vasospasm
Vasospasm up vs No vasospasm
Vasospasm up vs No vasospasm
Vasospasm up vs No vasospasm
Vasospasm up vs No vasospasm
Vasospasm down vs No vasospasm
Vasospasm down vs No vasospasm
Vasospasm up vs No vasospasm
Vasospasm down vs No vasospasm
Vasospasm down vs No vasospasm
Vasospasm up vs No vasospasm
Vasospasm up vs No vasospasm
Vasospasm down vs No vasospasm
Vasospasm up vs No vasospasm
Vasospasm up vs No vasospasm
Vasospasm up vs No vasospasm
Vasospasm down vs No vasospasm
Vasospasm down vs No vasospasm
Vasospasm up vs No vasospasm
Vasospasm up vs No vasospasm
Vasospasm up vs No vasospasm
Vasospasm up vs No vasospasm
Vasospasm up vs No vasospasm
Vasospasm up vs No vasospasm
Vasospasm up vs No vasospasm
Vasospasm up vs No vasospasm
Vasospasm up vs No vasospasm
Vasospasm up vs No vasospasm
Vasospasm up vs No vasospasm
Vasospasm up vs No vasospasm
Vasospasm down vs No vasospasm
Vasospasm up vs No vasospasm

Vasospasm up vs No vasospasm
Vasospasm down vs No vasospasm
Vasospasm down vs No vasospasm
Vasospasm up vs No vasospasm
Vasospasm up vs No vasospasm
Vasospasm up vs No vasospasm
Vasospasm up vs No vasospasm
Vasospasm up vs No vasospasm
Vasospasm up vs No vasospasm
Vasospasm down vs No vasospasm
Vasospasm up vs No vasospasm
Vasospasm up vs No vasospasm
Vasospasm up vs No vasospasm
Vasospasm up vs No vasospasm
Vasospasm up vs No vasospasm
Vasospasm up vs No vasospasm
Vasospasm up vs No vasospasm
Vasospasm up vs No vasospasm
Vasospasm up vs No vasospasm
Vasospasm up vs No vasospasm
Vasospasm up vs No vasospasm
Vasospasm up vs No vasospasm
Vasospasm down vs No vasospasm
Vasospasm up vs No vasospasm
Vasospasm up vs No vasospasm
Vasospasm up vs No vasospasm
Vasospasm up vs No vasospasm
Vasospasm up vs No vasospasm
Vasospasm down vs No vasospasm
Vasospasm up vs No vasospasm
Vasospasm up vs No vasospasm
Vasospasm up vs No vasospasm
Vasospasm down vs No vasospasm
Vasospasm down vs No vasospasm
Vasospasm up vs No vasospasm
Vasospasm up vs No vasospasm
Vasospasm down vs No vasospasm
Vasospasm up vs No vasospasm
Vasospasm up vs No vasospasm
Vasospasm up vs No vasospasm
Vasospasm down vs No vasospasm
Vasospasm down vs No vasospasm
Vasospasm up vs No vasospasm
Vasospasm up vs No vasospasm
Vasospasm up vs No vasospasm
Vasospasm up vs No vasospasm
Vasospasm up vs No vasospasm

Vasospasm up vs No vasospasm
Vasospasm up vs No vasospasm
Vasospasm up vs No vasospasm
Vasospasm up vs No vasospasm
Vasospasm up vs No vasospasm
Vasospasm up vs No vasospasm
Vasospasm down vs No vasospasm
Vasospasm up vs No vasospasm
Vasospasm up vs No vasospasm
Vasospasm up vs No vasospasm
Vasospasm up vs No vasospasm
Vasospasm up vs No vasospasm
Vasospasm down vs No vasospasm
Vasospasm up vs No vasospasm
Vasospasm up vs No vasospasm
Vasospasm up vs No vasospasm
Vasospasm down vs No vasospasm
Vasospasm up vs No vasospasm
Vasospasm down vs No vasospasm
Vasospasm up vs No vasospasm
Vasospasm up vs No vasospasm
Vasospasm up vs No vasospasm
Vasospasm up vs No vasospasm
Vasospasm up vs No vasospasm
Vasospasm up vs No vasospasm
Vasospasm up vs No vasospasm
Vasospasm up vs No vasospasm
Vasospasm up vs No vasospasm
Vasospasm up vs No vasospasm
Vasospasm down vs No vasospasm
Vasospasm down vs No vasospasm
Vasospasm up vs No vasospasm
Vasospasm up vs No vasospasm
Vasospasm up vs No vasospasm
Vasospasm down vs No vasospasm
Vasospasm up vs No vasospasm
Vasospasm up vs No vasospasm
Vasospasm up vs No vasospasm
Vasospasm up vs No vasospasm
Vasospasm up vs No vasospasm
Vasospasm up vs No vasospasm
Vasospasm up vs No vasospasm
