# Supplementary material for: mRNA Expression Profiles from Whole Blood Associated with Vasospasm in Patients with Subarachnoid Hemorrhage
Source: Neurocrit Care. 2019 Oct 8;33(1):82–9. doi: 10.1007/s12028-019-00861-x (PMC7392923; doi:10.1007/s12028-019-00861-x)
Supplement: Supplementary file 2 — Supplementary material 2 (PDF 344 kb) [file 12028_2019_861_MOESM2_ESM.pdf]

Gene Symbol	transcript_cluster_id	Probeset ID	p-value (Vasospasm vs. No vasospasm)
LMO1	3361672	3361727	2.49E-05
GLDN	3593931	3593961	5.16E-05
HOXB6	3761395	3761398	5.51E-05
ESPL1	3415857	3415861	5.61E-05
DNAH10	3436117	3436158	6.05E-05
PHOX2B	2767159	2767171	6.19E-05
SGTA	3846011	3846013	6.21E-05
FAM55C	2634058	2634063	6.42E-05
MGC2752	3844297	3844299	7.63E-05
RAB11FIP3	3642875	3642881	8.92E-05
C5orf38	2799509	2799510	9.13E-05
KRT25	3756447	3756461	9.80E-05
CSNK1G1	3629012	3629055	9.97E-05
CDH15	3673892	3673906	1.06E-04
ABCA7	3815416	3815489	1.12E-04
IL24	2377035	2377050	1.27E-04
PPM1F	3954294	3954298	1.38E-04
ECD	3294242	3294264	1.43E-04
ARHGEF2	2437801	2437837	1.52E-04
E2F1	3903146	3903157	1.53E-04
TEX13B	4017501	4017506	1.58E-04
TMEM67	3107242	3107263	1.65E-04
CBFA2T2	3882533	3882603	1.66E-04
PPP2R4	3190939	3190968	1.87E-04
CLN6	3630701	3630716	1.90E-04
NT5C3L	3757399	3757420	1.92E-04
EXT1	3150060	3150162	1.99E-04
AKR7L	2399687	2399700	2.13E-04
HRC	3867708	3867710	2.13E-04
POLR3H	3961981	3961989	2.15E-04
GIT1	3751541	3751569	2.22E-04
MYOM1	3796428	3796507	2.31E-04
	4053986	4053993	2.37E-04
ACACB	3430959	3431035	2.47E-04
GRASP	3415193	3415211	2.47E-04
ProSAPiP1	3895274	3895286	2.47E-04
C1orf127	2396362	2396373	2.54E-04
NR5A2	2374126	2374165	2.56E-04
UGGT2	3521484	3521601	2.59E-04
ORC5L	3065963	3065969	2.64E-04
ADAM7	3090326	3090337	2.70E-04
PDE2A	3381150	3381160	2.70E-04
NPR2	3168160	3168187	2.70E-04
PRPH	3413852	3413858	2.72E-04
DNAH3	3683879	3683918	2.75E-04
FBXL4	2966078	2966124	2.83E-04

RPTOR	3737488	3737627	2.85E-04
MYO1C	3740201	3740232	2.92E-04
BATF	3544605	3544623	2.95E-04
DACT2	2985497	2985513	2.98E-04
	2867145	2867206	3.09E-04
EVC2	2758978	2759034	3.16E-04
KPTN	3866579	3866594	3.18E-04
LACRT	3456878	3456879	3.28E-04
SPOCK3	2792647	2792670	3.29E-04
CHD9	3660858	3661003	3.29E-04
CEP135	2727976	2727994	3.33E-04
CCDC85B	3335719	3335725	3.34E-04
PIBF1	3493448	3493520	3.40E-04
PSIP1	3199790	3199820	3.43E-04
GPR35	2535927	2535931	3.51E-04
SLC15A1	3522327	3522374	3.54E-04
AKAP14	3989006	3989009	3.60E-04
KIF22	3655628	3655630	3.73E-04
SNAI1	3888522	3888529	3.76E-04
PTPRF	2333318	2333344	3.76E-04
GMPPA	2528620	2528635	3.80E-04
PHACTR3	3891530	3891600	3.87E-04
HSPA14	3236395	3236436	3.89E-04
SPSB3	3676134	3676136	3.89E-04
INTS8	3107661	3107704	3.93E-04
IDH3G	4026722	4026736	3.93E-04
PPA2	2780522	2780593	3.95E-04
FAM122C	3991814	3991834	3.96E-04
SLC28A3	3212420	3212442	4.01E-04
C20orf196	3875108	3875157	4.09E-04
NR1H2	3839276	3839299	4.17E-04
C8orf44	3101765	3101781	4.27E-04
GPR126	2928461	2928529	4.33E-04
ATP2C1	2642325	2642388	4.35E-04
SMOC1	3542275	3542354	4.36E-04
FAM134C	3757990	3758006	4.39E-04
NEB	2581000	2581017	4.42E-04
SLC12A8	2693014	2693025	4.46E-04
ZFYVE21	3553947	3553953	4.53E-04
MRPL17	3361116	3361129	4.59E-04
SRGAP3	2662087	2662104	4.59E-04
CDC42BPB	3580498	3580524	4.60E-04
SH2D1A	3989826	3989844	4.67E-04
DDR2	2364231	2364285	4.69E-04
C1orf66	2361697	2361710	4.71E-04
ITPR2	3448152	3448325	4.74E-04
ZNF397	3784468	3784495	4.77E-04

ZNF266	3849688	3849692	4.81E-04
GANAB	3375951	3375966	4.85E-04
FLJ34690	3710804	3710806	4.85E-04
KNTC1	3435362	3435388	4.97E-04
DENND4A	3629811	3629860	5.00E-04
ZNF236	3794458	3794540	5.04E-04
DNAJB7	3961699	3961700	5.37E-04
SIM2	3920171	3920213	5.39E-04
SH3BGR	3921442	3921449	5.41E-04
CACNA1B	3195568	3195661	5.43E-04
NUBPL	3531355	3531358	5.44E-04
TLL1	2750753	2750760	5.53E-04
PARD3B	2524016	2524018	5.57E-04
NKX2-1	3561381	3561412	5.64E-04
MAGI2	3058209	3058245	5.72E-04
RCAN1	3930235	3930278	5.73E-04
SLC17A8	3428190	3428202	5.75E-04
RP13-102H20.1	3990927	3990942	5.84E-04
COL17A1	3305081	3305086	5.87E-04
LAMA5	3913018	3913111	5.90E-04
TRO	3978579	3978594	5.96E-04
TNS1	2599153	2599246	5.97E-04
UBR3	2514658	2514714	6.00E-04
C3	3848039	3848043	6.04E-04
4-Sep	3764527	3764559	6.05E-04
IRX1	2799758	2799776	6.06E-04
GRIN2D	3837759	3837781	6.14E-04
LNX2	3507003	3507015	6.17E-04
AKNA	3221916	3221970	6.19E-04
RAPH1	2595560	2595573	6.29E-04
TMEM132D	3478068	3478151	6.30E-04
ALS2CR8	2523419	2523447	6.36E-04
ZMAT4	3132616	3132697	6.38E-04
VARS	2949380	2949411	6.39E-04
FASN	3774635	3774666	6.43E-04
ABCA3	3676763	3676822	6.43E-04
MTFR1	3101385	3101397	6.55E-04
STAG3L4	3006133	3006147	6.63E-04
METRN	3643333	3643337	6.74E-04
CACNG7	3841134	3841148	6.75E-04
SPPL2B	3816424	3816457	6.85E-04
POU2F2	3863435	3863450	6.93E-04
CENPE	2780172	2780219	6.98E-04
GPR177	2417390	2417401	7.02E-04
SCGB3A1	2890741	2890755	7.05E-04
CNDP2	3793760	3793782	7.10E-04
NEGR1	2418078	2418080	7.12E-04

DTNBP1	2943236	2943246	7.16E-04
RNF17	3482017	3482024	7.33E-04
MKNK2	3845647	3845664	7.38E-04
METTL5	2586348	2586369	7.40E-04
ANK3	3290875	3291019	7.44E-04
C5orf33	2853388	2853395	7.45E-04
CLK1	2594497	2594510	7.47E-04
ADAMTSL1	3163982	3164038	7.48E-04
TRRAP	3014411	3014412	7.52E-04
RICH2	3710870	3710873	7.72E-04
UGT1A10	2533019	2533039	7.73E-04
INPP5K	3740264	3740272	7.78E-04
CASK	4005859	4005903	7.80E-04
GLT8D1	2676319	2676326	7.82E-04
SENP7	2686646	2686668	7.84E-04
KIAA1033	3429754	3429816	7.85E-04
KDELR3	3945314	3945329	7.85E-04
NUP210L	2436467	2436480	7.93E-04
MGC39372	2939014	2939016	8.04E-04
RYR1	3832457	3832585	8.05E-04
SUMF1	2660800	2660889	8.06E-04
SCLY	2534615	2534619	8.09E-04
TP53BP1	3621194	3621208	8.10E-04
TRPM1	3616044	3616045	8.14E-04
PLCB4	3875908	3875943	8.16E-04
C20orf103	3876084	3876093	8.19E-04
PLXNB1	2673181	2673201	8.22E-04
COL12A1	2961177	2961257	8.24E-04
KRT85	3455309	3455324	8.32E-04
SLC12A3	3662333	3662359	8.34E-04
LARS	2879927	2879970	8.34E-04
C3orf64	2681114	2681126	8.56E-04
PROM2	2493943	2493964	8.57E-04
LOC84931	2573326	2573330	8.59E-04
PKN1	3822723	3822744	8.61E-04
PHF8	4009506	4009527	8.62E-04
SLC12A5	3887241	3887251	8.68E-04
C9orf68	3197231	3197247	8.70E-04
ARAP2	2765590	2765623	8.79E-04
MAMLD1	3994710	3994745	8.81E-04
SLC25A32	3147726	3147782	8.83E-04
KIAA1984	3194635	3194680	8.86E-04
PCNXL2	2461037	2461092	8.99E-04
RP13-102H20.1	3990927	3990951	9.03E-04
CROCCL2	2398193	2398202	9.05E-04
SHROOM3	2732068	2732126	9.09E-04
C10orf79	3305198	3305226	9.09E-04

DHX57	2549007	2549035	9.11E-04
ARHGAP30	2440549	2440564	9.20E-04
LY6K	3119213	3119216	9.22E-04
SLC2A4RG	3893673	3893691	9.25E-04
TMC1	3174643	3174683	9.38E-04
TLR10	2766192	2766193	9.39E-04
ABCF3	2655511	2655512	9.40E-04
NOTCH3	3853108	3853138	9.41E-04
LMBR1	3081624	3081635	9.42E-04
OTOP2	3734517	3734529	9.46E-04
DCLRE1C	3278977	3279006	9.46E-04
PIP5K1C	3846316	3846317	9.48E-04
ZMYND19	3231157	3231160	9.50E-04
RAPGEF3	3452690	3452699	9.54E-04
DYNC1H1	3552847	3552892	9.57E-04
ADCY6	3453252	3453253	9.66E-04
B3GALT5	3921490	3921492	9.67E-04
ZZEF1	3741875	3741881	9.71E-04
TWSG1	3778372	3778373	9.75E-04
DCBLD2	2686023	2686025	9.78E-04
ZSCAN18	3872678	3872700	9.78E-04
PRKAB1	3434142	3434148	9.81E-04
ZNF267	3657367	3657378	9.85E-04
CACNA1C	3400730	3400930	9.97E-04
SLC5A10	3713627	3713663	9.98E-04
RNGTT	2963929	2964002	1.00E-03
DBH	3192820	3192879	1.01E-03
RANBP2	2499158	2499187	1.02E-03
BRWD1	3932148	3932158	1.02E-03
RGS5	2441386	2441394	1.02E-03
SCTR	2573112	2573151	1.02E-03
TAPT1	2761941	2761947	1.02E-03
USP5	3402899	3402901	1.03E-03
TSGA10	2566586	2566615	1.03E-03
ZZZ3	2419046	2419060	1.03E-03
PDZRN4	3411810	3411878	1.03E-03
FAM98C	3832435	3832453	1.03E-03
REM1	3881261	3881271	1.04E-03
MAP3K7IP2	2930592	2930649	1.05E-03
COL4A6	4017538	4017550	1.05E-03
ZNF180	3864921	3864922	1.06E-03
GLT8D3	3451246	3451260	1.06E-03
DLEC1	2617477	2617559	1.06E-03
POLG	3638337	3638378	1.06E-03
GDF5	3903952	3903954	1.06E-03
C2orf84	2473026	2473078	1.07E-03
EHD1	3377226	3377228	1.07E-03

ATP4B	3526655	3526670	1.08E-03
ZFYVE26	3569441	3569476	1.08E-03
PNCK	4026624	4026635	1.09E-03
ZNF575	3835085	3835100	1.09E-03
BARHL1	3192495	3192503	1.10E-03
CDC14A	2348896	2348899	1.11E-03
ELMOD2	2745067	2745090	1.13E-03
FAM160A2	3360772	3360784	1.13E-03
LXN	2702724	2702733	1.14E-03
LRRN4	3896594	3896603	1.14E-03
VGLL3	2684851	2684853	1.15E-03
KRT18	3415576	3415581	1.15E-03
CYP4F22	3823210	3823217	1.16E-03
STAR	3131819	3131836	1.17E-03
KCNK6	3832292	3832294	1.17E-03
BCL2	3811339	3811362	1.17E-03
OGFR	3892941	3892964	1.18E-03
CPAMD8	3854132	3854160	1.21E-03
ZNF417	3872521	3872529	1.21E-03
ABCA2	3230610	3230660	1.21E-03
LEPRE1	2409004	2409048	1.22E-03
TXNRD2	3952880	3952885	1.23E-03
AP3B2	3636216	3636237	1.23E-03
CACNA1C	3400730	3400807	1.23E-03
IFT88	3480411	3480479	1.23E-03
DNHD1	3318731	3318758	1.24E-03
TMEM115	2675304	2675308	1.25E-03
NCRNA00051	3119000	3119003	1.25E-03
ATP5L2	3962560	3962565	1.25E-03
LZTS2	3260957	3260965	1.27E-03
TMC3	3635578	3635608	1.28E-03
CAPN7	2612278	2612312	1.28E-03
TMF1	2681157	2681186	1.28E-03
WDR35	2542651	2542695	1.29E-03
KCNS2	3108782	3108786	1.30E-03
CASC1	3447798	3447816	1.31E-03
PLSCR4	2699623	2699661	1.31E-03
AIFM2	3293187	3293203	1.32E-03
FUK	3667241	3667262	1.32E-03
PDZD2	2805232	2805325	1.32E-03
NOL6	3203582	3203608	1.33E-03
ACRBP	3442150	3442158	1.33E-03
NOC3L	3301011	3301030	1.33E-03
NRXN2	3376914	3376969	1.33E-03
C8G	3194969	3194983	1.33E-03
RASGEF1B	2775259	2775286	1.34E-03
ABHD8	3854349	3854351	1.34E-03

MTSS1L	3697183	3697208	1.35E-03
ICAM5	3820469	3820482	1.35E-03
TNS3	3049522	3049569	1.36E-03
SMO	3023350	3023364	1.38E-03
LCP2	2886595	2886623	1.38E-03
MFSD6	2520138	2520146	1.39E-03
TRPM8	2533227	2533272	1.39E-03
TTC39A	2412312	2412320	1.39E-03
AARS2	2955118	2955147	1.39E-03
FLJ43860	3156655	3156710	1.40E-03
UNC13B	3167731	3167776	1.41E-03
TACC2	3268059	3268161	1.41E-03
MAPK3	3687494	3687497	1.41E-03
BRWD1	3932148	3932159	1.42E-03
ROBO1	2683763	2683792	1.42E-03
SLC37A1	3922664	3922713	1.42E-03
ESPL1	3415857	3415904	1.43E-03
LRRC4C	3370269	3370272	1.43E-03
APOBEC1	3442752	3442763	1.43E-03
C6orf145	2939469	2939470	1.44E-03
PCDHGA7	2832533	2832597	1.44E-03
RNASE7	3527831	3527835	1.46E-03
BTG3	3926080	3926094	1.47E-03
MAGOHB	3444195	3444196	1.47E-03
BRDT	2346575	2346596	1.48E-03
KIAA1468	3791168	3791202	1.48E-03
UMOD	3683549	3683553	1.48E-03
SCML2	4001369	4001379	1.49E-03
TP53I11	3371003	3371031	1.49E-03
GGT5	3955185	3955200	1.49E-03
CLMN	3577940	3577949	1.49E-03
SPINK5	2834503	2834526	1.49E-03
RYR1	3832457	3832563	1.49E-03
HPRT1	3991698	3991716	1.49E-03
C20orf71	3882369	3882377	1.50E-03
RNASEL	2447124	2447134	1.50E-03
SCYL1	3335267	3335289	1.51E-03
GPLD1	2945518	2945546	1.51E-03
AARS2	2955118	2955140	1.52E-03
KIF15	2620256	2620275	1.52E-03
ZNF7	3120917	3120951	1.52E-03
STK32B	2716713	2716766	1.52E-03
SPATA2L	3704928	3704932	1.53E-03
KIAA1524	2687979	2687996	1.53E-03
FLJ30679	3672640	3672643	1.53E-03
WDR91	3074260	3074280	1.54E-03
PAK6	3589756	3589772	1.54E-03

COL16A1	2404546	2404569	1.55E-03
NEK1	2793221	2793239	1.55E-03
SNTG1	3097701	3097820	1.55E-03
MAD2L1	2783715	2783743	1.56E-03
CCDC150	2521278	2521304	1.59E-03
DYNC1H1	3552847	3552909	1.60E-03
CAPN7	2612278	2612296	1.60E-03
	3642200	3642256	1.60E-03
SNX14	2963313	2963341	1.60E-03
ESYT3	2644619	2644654	1.62E-03
ZNF45	3864725	3864734	1.62E-03
USH2A	2455699	2455708	1.62E-03
CFP	4007164	4007176	1.62E-03
SLC4A3	2528774	2528810	1.63E-03
ARHGAP6	3999568	3999580	1.63E-03
SMARCA5	2745646	2745662	1.63E-03
GPR116	2955863	2955877	1.63E-03
OR2D3	3318982	3318985	1.63E-03
DTNBP1	2943236	2943245	1.63E-03
AR	3979912	3979925	1.64E-03
ALDOC	3750767	3750781	1.64E-03
ZNF276	3674349	3674369	1.64E-03
HOOK1	2338625	2338636	1.64E-03
MAP6	3382410	3382422	1.65E-03
BMP7	3910980	3910996	1.65E-03
CCNY	3242425	3242429	1.65E-03
CCM2	3000167	3000193	1.65E-03
CLDN15	3064574	3064589	1.65E-03
DRP2	3984702	3984737	1.66E-03
CCDC62	3435515	3435542	1.66E-03
CEP192	3779817	3779928	1.66E-03
TET1	3249886	3249895	1.67E-03
OMA1	2414558	2414781	1.67E-03
ALDH2	3432090	3432101	1.67E-03
C14orf177	3551029	3551037	1.67E-03
TNKS1BP1	3373675	3373710	1.68E-03
SLC1A1	3160658	3160687	1.69E-03
SNX20	3691193	3691204	1.69E-03
PPOX	2363484	2363488	1.70E-03
DENND4C	3164221	3164270	1.70E-03
C1orf63	2402111	2402121	1.71E-03
CFH	2373336	2373344	1.71E-03
SCAF1	3838757	3838777	1.71E-03
MACF1	2331213	2331352	1.72E-03
KCTD20	2905069	2905070	1.72E-03
PEX5	3403299	3403305	1.73E-03
MAPK15	3119792	3119814	1.73E-03

INO80	3619773	3619866	1.73E-03
TYMP	3966000	3966002	1.73E-03
FAM188B	2995491	2995555	1.73E-03
TRAM2	2957227	2957231	1.73E-03
PRDM2	2321238	2321289	1.74E-03
CACNA1B	3195568	3195701	1.75E-03
NR5A1	3225058	3225070	1.75E-03
TRPM5	3359267	3359277	1.76E-03
DEAF1	3358262	3358290	1.77E-03
TRRAP	3014411	3014435	1.77E-03
SLC43A3	3373845	3373855	1.78E-03
PHKB	3659156	3659212	1.78E-03
PADI2	2398820	2398841	1.78E-03
SARDH	3228813	3228846	1.79E-03
PBRM1	2676219	2676289	1.80E-03
MARCKS	2922215	2922225	1.81E-03
RYR2	2387126	2387322	1.81E-03
MGC39372	2939014	2939015	1.81E-03
LRRC6	3154136	3154159	1.81E-03
ZNF385B	2590017	2590028	1.82E-03
GCOM1	3595441	3595442	1.82E-03
BCOR	4005392	4005429	1.83E-03
LRRC40	2417737	2417740	1.83E-03
PITPNM2	3475926	3475964	1.84E-03
BFSP1	3899111	3899147	1.84E-03
RARA	3720921	3720951	1.84E-03
SGPP2	2529421	2529461	1.84E-03
SH3TC2	2880679	2880714	1.86E-03
CCDC126	2992963	2992983	1.87E-03
ANXA13	3151719	3151726	1.87E-03
PSG1	3863761	3863789	1.88E-03
OVOL1	3335571	3335574	1.89E-03
DIS3	3493391	3493439	1.89E-03
DDX59	2450416	2450438	1.90E-03
ERLEC1	2482230	2482231	1.90E-03
CSF1R	2881187	2881191	1.90E-03
SLC25A19	3770721	3770723	1.91E-03
GSTA2	2957314	2957328	1.91E-03
UGT3A2	2853325	2853342	1.92E-03
CHD1L	2356721	2356755	1.92E-03
MCM5	3944147	3944171	1.92E-03
CLEC10A	3743306	3743324	1.93E-03
C3orf17	2688882	2688908	1.94E-03
ISL1	2808931	2808947	1.95E-03
MGAT5	2506903	2506988	1.96E-03
CTNS	3706700	3706709	1.97E-03
ZNF433	3851150	3851186	1.97E-03

KIAA0664	3741171	3741178	1.98E-03
KCNN2	2824581	2824635	1.98E-03
PRUNE	2358623	2358633	1.99E-03
SPAG4	3883441	3883444	1.99E-03
ABCC3	3726691	3726731	1.99E-03
DZIP3	2635263	2635326	1.99E-03
POL3S	3688197	3688218	2.00E-03
EPC2	2509832	2509885	2.00E-03
SCIN	2990404	2990426	2.00E-03
SLC38A5	4007437	4007460	2.00E-03
FOXC1	2891768	2891781	2.01E-03
DDX51	3478957	3478986	2.01E-03
CUL5	3347549	3347580	2.01E-03
FBXW12	2621647	2621661	2.02E-03
SOX4	2897899	2897914	2.03E-03
CPB2	3512843	3512848	2.03E-03
ZCCHC3	3873102	3873106	2.03E-03
ANK3	3290875	3290917	2.03E-03
RAB11FIP3	3642875	3642929	2.04E-03
KCNN2	2824581	2824626	2.04E-03
SLC48A1	3413212	3413231	2.04E-03
RAD51	3590086	3590099	2.05E-03
ANKRD11	3704717	3704781	2.05E-03
C1orf63	2402111	2402115	2.06E-03
TROAP	3413875	3413880	2.07E-03
KRT25	3756447	3756450	2.07E-03
SLC17A3	2946106	2946118	2.08E-03
TBX19	2366184	2366209	2.08E-03
NLRP14	3319018	3319020	2.08E-03
COQ4	3190339	3190347	2.09E-03
EPHB2	2324919	2324962	2.09E-03
BAT2	2902463	2902495	2.09E-03
ITIH1	2624147	2624158	2.09E-03
PON1	3061942	3061944	2.09E-03
NEK5	3514804	3514840	2.10E-03
PIBF1	3493448	3493477	2.10E-03
SOX2	2654454	2654505	2.11E-03
NEK1	2793221	2793237	2.11E-03
MMP15	3663074	3663095	2.11E-03
TEF	3946817	3946834	2.12E-03
ZFAND3	2905664	2905668	2.12E-03
GPR152	3337109	3337110	2.12E-03
BPHL	2892393	2892400	2.13E-03
RNASEL	2447124	2447128	2.13E-03
PLA2G6	3960388	3960400	2.13E-03
PHKB	3659156	3659233	2.14E-03
CAPN11	2908371	2908401	2.15E-03

PEX5L	2707045	2707051	2.15E-03
BMP4	3565206	3565209	2.15E-03
C1orf97	2378710	2378713	2.16E-03
PCSK2	3877892	3877950	2.16E-03
TRPM2	3923702	3923708	2.17E-03
SLC44A2	3820612	3820626	2.17E-03
SYTL4	4015440	4015449	2.17E-03
C10orf79	3305198	3305247	2.17E-03
ADH4	2779124	2779136	2.17E-03
POLR1B	2500838	2500853	2.18E-03
ACSS1	3901696	3901713	2.18E-03
NCRNA00164	2576788	2576808	2.18E-03
TGFB1	3863021	3863040	2.18E-03
LMBRD1	2960010	2960031	2.19E-03
SETMAR	2608419	2608446	2.20E-03
C20orf43	3890218	3890219	2.20E-03
ALDH3A2	3714068	3714099	2.20E-03
CDC42BPG	3377177	3377217	2.22E-03
ZNF695	2465395	2465396	2.23E-03
PARD3	3284596	3284604	2.23E-03
PYGO1	3625440	3625449	2.23E-03
PLEKHA7	3364525	3364582	2.23E-03
HUNK	3917938	3917950	2.23E-03
TNK1	3708528	3708532	2.24E-03
USP8	3593652	3593701	2.24E-03
SEC14L1	3735752	3735775	2.24E-03
APOH	3767709	3767723	2.24E-03
PDGFB	3961068	3961089	2.25E-03
ASRGL1	3333443	3333461	2.25E-03
SF3B1	2593670	2593674	2.26E-03
UGT3A1	2853293	2853319	2.26E-03
LOXHD1	3806366	3806399	2.27E-03
SCN8A	3414969	3414983	2.27E-03
ACAN	3607332	3607370	2.28E-03
P2RX2	3439132	3439140	2.29E-03
ASB14	2677922	2677932	2.29E-03
C17orf53	3722770	3722792	2.29E-03
FMNL3	3454006	3454024	2.30E-03
BMP6	2893895	2893900	2.30E-03
TNC	3222170	3222177	2.31E-03
FBXW5	3230530	3230534	2.31E-03
NRD1	2412529	2412586	2.32E-03
CASK	4005859	4005872	2.32E-03
NEK1	2793221	2793228	2.32E-03
POLA1	3972093	3972120	2.32E-03
KLHL14	3803418	3803464	2.32E-03
LUC7L3	3726772	3726781	2.33E-03

CCDC142	2560149	2560158	2.33E-03
PLK2	2858023	2858046	2.34E-03
PREX2	3102096	3102192	2.35E-03
MAL2	3113180	3113183	2.35E-03
ANKRD53	2487963	2487973	2.36E-03
DOCK7	2415910	2415975	2.36E-03
FLNA	4027176	4027189	2.36E-03
BXDC2	2806231	2806237	2.36E-03
RAI2	4001223	4001240	2.36E-03
GRIN2D	3837759	3837767	2.37E-03
LPHN2	2343823	2343945	2.37E-03
LRRC6	3154136	3154155	2.38E-03
CPEB2	2719361	2719405	2.38E-03
JAG1	3897505	3897536	2.39E-03
PDCD11	3262198	3262215	2.39E-03
IRAK1	4027009	4027040	2.39E-03
LNX1	2769346	2769350	2.39E-03
SCNN1D	2315674	2315697	2.39E-03
SMC6	2541944	2541946	2.39E-03
LIPG	3787855	3787871	2.40E-03
P4HA1	3294159	3294180	2.40E-03
RASGRF2	2817941	2817944	2.40E-03
ERLIN1	3303255	3303292	2.40E-03
TEX13A	4016955	4016976	2.41E-03
MED24	3755976	3756031	2.41E-03
PPP1R8	2327259	2327279	2.41E-03
SCN1A	2585322	2585334	2.42E-03
RNF133	3070499	3070505	2.42E-03
SUPT16H	3556323	3556325	2.42E-03
NUP210L	2436467	2436502	2.43E-03
PLXND1	2694817	2694909	2.43E-03
PPP1R7	2536183	2536193	2.43E-03
SCN1A	2585322	2585354	2.43E-03
ADAM11	3723128	3723140	2.44E-03
CHN1	2587961	2587985	2.44E-03
GRM1	2929571	2929650	2.45E-03
SCD5	2775735	2775739	2.45E-03
WDR35	2542651	2542702	2.45E-03
HKDC1	3250237	3250239	2.45E-03
DDAH1	2420832	2420853	2.46E-03
C16orf14	3643114	3643130	2.46E-03
PRMT2	3924783	3924804	2.46E-03
ARHGAP6	3999568	3999587	2.47E-03
QTRT1	3820727	3820740	2.48E-03
SAPS3	3337618	3337680	2.49E-03
CENPE	2780172	2780220	2.49E-03
ZC3H18	3673515	3673524	2.50E-03

HCN1	2855963	2855968	2.51E-03
CCDC65	3413643	3413655	2.51E-03
C1QTNF5	3394330	3394338	2.52E-03
ICOSLG	3934407	3934430	2.52E-03
C7orf16	2995811	2995824	2.52E-03
TMEM71	3154185	3154200	2.52E-03
SLC28A3	3212420	3212441	2.53E-03
PNPLA6	3818897	3818962	2.53E-03
CTSG	3558347	3558357	2.53E-03
SCNN1D	2315674	2315729	2.53E-03
PDZD4	4026757	4026763	2.53E-03
BZRAP1	3764289	3764332	2.55E-03
DLEC1	2617477	2617536	2.55E-03
ATP4A	3859832	3859861	2.57E-03
STAG1	2696802	2696844	2.57E-03
DHX9	2370991	2371037	2.58E-03
PDS5A	2766588	2766623	2.58E-03
MREG	2598496	2598506	2.58E-03
FRK	2970897	2970900	2.59E-03
RLF	2331771	2331805	2.59E-03
PFKFB4	2673312	2673317	2.59E-03
ATL3	3376560	3376574	2.59E-03
PKHD1L1	3111561	3111644	2.59E-03
ADORA2B	3711869	3711870	2.59E-03
FAM148A	3596894	3596904	2.59E-03
ZNF507	3828887	3828930	2.60E-03
DNAH2	3709010	3709026	2.60E-03
SCMH1	2408499	2408504	2.61E-03
GPR52	2368180	2368183	2.62E-03
ZKSCAN3	2900497	2900502	2.62E-03
TRPV4	3470927	3470930	2.62E-03
ARID3A	3815278	3815280	2.62E-03
LTBP3	3377669	3377694	2.64E-03
MYO10	2850071	2850163	2.64E-03
BNIPL	2358646	2358655	2.64E-03
CACNG5	3732049	3732052	2.64E-03
TTRAP	2945645	2945648	2.65E-03
AGPAT5	3083936	3083966	2.65E-03
ADCY7	3659966	3659988	2.65E-03
UGGT1	2504883	2504935	2.65E-03
UACA	3631397	3631402	2.66E-03
CENPB	3895722	3895723	2.66E-03
CDH17	3144859	3144887	2.67E-03
AGPAT1	2949801	2949808	2.67E-03
HMGXB4	3944046	3944082	2.68E-03
RHOT1	3717539	3717551	2.68E-03
NEDD9	2941784	2941802	2.69E-03

MYO15B	3734966	3735083	2.70E-03
PLA2G2F	2323882	2323886	2.70E-03
ADAMTSL3	3605395	3605491	2.71E-03
CDK6	3061319	3061327	2.71E-03
C11orf42	3318595	3318603	2.71E-03
LYPD6	2510056	2510088	2.73E-03
AIPL1	3743038	3743064	2.73E-03
SIX5	3865618	3865625	2.73E-03
AHI1	2975385	2975392	2.73E-03
AFAP1L2	3307851	3307869	2.74E-03
RAB1B	3336074	3336089	2.75E-03
CD5L	2439138	2439157	2.75E-03
MED16	3844822	3844844	2.75E-03
A2BP1	3646613	3646903	2.76E-03
C2orf56	2477372	2477377	2.76E-03
UBE2A	3988874	3988886	2.77E-03
LILRB4	3841621	3841632	2.77E-03
FLJ42875	2392945	2392974	2.77E-03
ACTN2	2386943	2386991	2.77E-03
TLR10	2766192	2766197	2.77E-03
DZIP1	3521372	3521415	2.77E-03
MUSK	3184710	3184738	2.78E-03
PLSCR1	2699726	2699767	2.78E-03
SNORA66	2346863	2346909	2.78E-03
MTERFD1	3145586	3145588	2.78E-03
CACNA1I	3945942	3946025	2.79E-03
CPVL	3043648	3043693	2.79E-03
CHD5	2394478	2394532	2.79E-03
PTPRB	3461795	3461844	2.79E-03
TMEM200B	2403707	2403714	2.79E-03
BCL7C	3688038	3688054	2.80E-03
GRIK2	2918982	2919062	2.80E-03
PKN2	2345617	2345698	2.81E-03
FBN2	2874371	2874469	2.82E-03
ZNF503	3295376	3295431	2.82E-03
SLC30A8	3112584	3112628	2.83E-03
PCCB	2644014	2644040	2.83E-03
DECR2	3642837	3642852	2.83E-03
C17orf50	3718775	3718785	2.84E-03
ZNF493	3826601	3826613	2.84E-03
SPTBN5	3620156	3620207	2.85E-03
PTPRO	3406329	3406379	2.86E-03
MYST1	3656855	3656862	2.86E-03
DTNBP1	2943236	2943244	2.87E-03
SNRNP48	2893847	2893861	2.87E-03
BBS9	2996321	2996337	2.87E-03
KIAA1033	3429754	3429782	2.87E-03

DLG5	3296386	3296430	2.87E-03
RNF7	2645690	2645696	2.87E-03
FAM47B	3973396	3973398	2.88E-03
LAMB3	2453793	2453809	2.88E-03
FCGBP	3862188	3862202	2.89E-03
PPIL4	2978876	2978913	2.89E-03
PTGES	3227070	3227085	2.89E-03
APLF	2486851	2486873	2.90E-03
DCAF5	3569926	3569953	2.90E-03
C11orf59	3380980	3380991	2.91E-03
GFRA1	3308241	3308341	2.91E-03
NKD2	2798952	2798985	2.91E-03
CNOT7	3125775	3125783	2.91E-03
ABHD12B	3535307	3535308	2.92E-03
ZC3H6	2500722	2500762	2.92E-03
CRISPLD2	3671935	3671948	2.93E-03
GRIK1	3928211	3928246	2.93E-03
PAPPA2	2368590	2368597	2.93E-03
HCN4	3632424	3632436	2.93E-03
TRPM6	3210013	3210095	2.93E-03
ING3	3021123	3021135	2.93E-03
FLT1	3507282	3507322	2.94E-03
BCL2	3811339	3811448	2.94E-03
IQCE	2987578	2987616	2.94E-03
LIPG	3787855	3787861	2.94E-03
ANKRA2	2862380	2862400	2.95E-03
INTS10	3088405	3088429	2.96E-03
KCTD17	3944637	3944645	2.96E-03
WDR75	2519756	2519785	2.96E-03
DCAF8	2440018	2440089	2.99E-03
TET3	2489071	2489105	2.99E-03
ATM	3347658	3347731	2.99E-03
BZRAP1	3764289	3764344	2.99E-03
ITGB8	2991860	2991899	3.00E-03
NTN5	3867287	3867296	3.01E-03
SLC26A4	3018605	3018612	3.01E-03
ETNK2	2451870	2451888	3.02E-03
SF4	3855660	3855690	3.03E-03
FLJ46010	2812591	2812620	3.03E-03
GPR98	2819779	2819846	3.04E-03
BRSK1	3842059	3842079	3.04E-03
NUDCD1	3148796	3148811	3.04E-03
WDR60	3034449	3034473	3.04E-03
PEX19	2440117	2440124	3.04E-03
TTC14	2654306	2654321	3.04E-03
PKP2	3450234	3450240	3.04E-03
GIN1	2869275	2869296	3.04E-03

SNAP25	3876245	3876282	3.05E-03
ATXN10	3948754	3948847	3.05E-03
RYR1	3832457	3832489	3.06E-03
SMARCD2	3766415	3766425	3.06E-03
CNPY4	3015276	3015285	3.06E-03
YEATS2	2655168	2655172	3.07E-03
STC1	3128046	3128074	3.07E-03
GRWD1	3837796	3837815	3.07E-03
PAPLN	3543539	3543562	3.07E-03
RNF32	3033728	3033754	3.07E-03
PTGES	3227070	3227086	3.07E-03
RB1CC1	3135184	3135210	3.07E-03
TMEM126B	3342983	3342998	3.08E-03
ANKRD32	2820622	2820683	3.08E-03
RAB20	3525498	3525522	3.09E-03
MAP4	2672966	2672969	3.09E-03
SHANK1	3868587	3868604	3.09E-03
SCUBE1	3962839	3962893	3.10E-03
SEMA6D	3592755	3592851	3.10E-03
FHL2	2568687	2568729	3.10E-03
STAG3	3015338	3015343	3.11E-03
CNNM1	3260265	3260311	3.11E-03
INPP5F	3267382	3267399	3.11E-03
SYT9	3319073	3319100	3.11E-03
ITIH5L	4009751	4009758	3.11E-03
CARD14	3737192	3737232	3.12E-03
	2494151	2494159	3.13E-03
NBR1	3722417	3722470	3.13E-03
KIRREL	2362089	2362139	3.13E-03
TMEM165	2727793	2727838	3.14E-03
TRPM4	3838317	3838328	3.14E-03
C15orf27	3602569	3602603	3.14E-03
EFR3B	2473376	2473406	3.14E-03
ZNF770	3617830	3617831	3.14E-03
FGF1	2879166	2879188	3.15E-03
ZNF395	3129304	3129333	3.15E-03
SLC8A3	3570373	3570379	3.16E-03
RNGTT	2963929	2964012	3.16E-03
EXO1	2388219	2388244	3.19E-03
RBL1	3904747	3904752	3.19E-03
KIAA0427	3787675	3787687	3.20E-03
SSH3	3336906	3336944	3.20E-03
LCP2	2886595	2886621	3.21E-03
PUS7L	3451670	3451689	3.21E-03
ZDHHC17	3423184	3423199	3.21E-03
LRRC6	3154136	3154151	3.21E-03
PCDH24	2842624	2842664	3.21E-03

PNLIPRP2	3265952	3265966	3.22E-03
NEDD9	2941784	2941793	3.22E-03
BCL7C	3688038	3688059	3.22E-03
MMAA	2746164	2746178	3.23E-03
NXF3	4016319	4016321	3.23E-03
BCL7C	3688038	3688060	3.24E-03
SCEL	3494629	3494683	3.25E-03
ABCC12	3690388	3690410	3.26E-03
ZNF721	2756404	2756420	3.26E-03
PPCDC	3602039	3602047	3.26E-03
LRP5	3337516	3337600	3.27E-03
CCT4	2555630	2555674	3.27E-03
MRPL13	3150797	3150819	3.27E-03
TBX2	3729834	3729878	3.27E-03
ADAMTS19	2827772	2827775	3.28E-03
HEMK1	2622859	2622890	3.28E-03
KIAA1712	2752243	2752258	3.28E-03
LOC84856	3243581	3243593	3.28E-03
HIP1	3057370	3057390	3.29E-03
ATP2C1	2642325	2642385	3.29E-03
UPK1B	2637831	2637844	3.30E-03
ZHX2	3113894	3113896	3.30E-03
FAM71E2	3871389	3871397	3.30E-03
RNF160	3927949	3927984	3.31E-03
RGS18	2372719	2372727	3.32E-03
AGGF1	2816563	2816583	3.32E-03
UNC45A	3608520	3608538	3.33E-03
TJP1	3615579	3615600	3.33E-03
CSPG5	2672821	2672824	3.33E-03
GLCCI1	2989537	2989653	3.33E-03
TJP1	3615579	3615619	3.33E-03
TEAD3	2951500	2951502	3.33E-03
CC2D1A	3822444	3822489	3.35E-03
ZNF317	3819880	3819900	3.36E-03
SERPINB12	3791815	3791837	3.36E-03
KLK10	3868828	3868838	3.36E-03
CEP350	2369843	2369870	3.36E-03
MUC4	2712236	2712276	3.36E-03
MED14	4005644	4005649	3.36E-03
CRYGS	2709402	2709407	3.36E-03
TCFL5	3913483	3913507	3.37E-03
TNFAIP8L3	3623948	3623951	3.37E-03
SNAP29	3937755	3937759	3.37E-03
FBXO8	2794356	2794357	3.37E-03
ESYT2	3082248	3082253	3.38E-03
C5orf34	2855614	2855626	3.39E-03
CLOCK	2769947	2769999	3.39E-03

PLCB2	3619326	3619344	3.39E-03
CNTROB	3709327	3709343	3.39E-03
SMC3	3263790	3263807	3.40E-03
IFNA5	3201255	3201261	3.40E-03
SLC22A9	3333831	3333860	3.40E-03
WDR64	2388148	2388203	3.42E-03
IL27RA	3822551	3822567	3.42E-03
FLT1	3507282	3507375	3.43E-03
CDC14A	2348896	2348934	3.43E-03
MCM5	3944147	3944174	3.43E-03
ERCC1	3865378	3865397	3.43E-03
MAP3K7IP2	2930592	2930650	3.44E-03
PRPF6	3893849	3893865	3.44E-03
SMARCA4	3820921	3820965	3.44E-03
ESCO1	3800779	3800799	3.44E-03
VWF	3441685	3441785	3.44E-03
GOLGA1	3225224	3225282	3.45E-03
MRGPRF	3379777	3379781	3.45E-03
NAPSA	3868400	3868435	3.45E-03
CTTN	3338552	3338613	3.45E-03
KLK2	3839563	3839570	3.45E-03
KIAA0391	3532393	3532426	3.45E-03
UNQ9374	2886535	2886539	3.46E-03
CKAP5	3371719	3371784	3.46E-03
DNAI1	3167427	3167431	3.46E-03
CUL9	2907754	2907791	3.46E-03
BRD8	2877257	2877291	3.47E-03
EPB41L1	3883690	3883773	3.47E-03
LILRA3	3870611	3870670	3.48E-03
SLC9A3	2845362	2845366	3.48E-03
ASCC3	2966636	2966723	3.48E-03
C20orf185	3882241	3882254	3.49E-03
LMO7	3494137	3494238	3.49E-03
PHGDH	2354634	2354690	3.49E-03
ZP3	3009441	3009448	3.49E-03
PTPRE	3270270	3270353	3.50E-03
MUPCDH	3358201	3358235	3.50E-03
TTK	2914777	2914782	3.52E-03
DENND5A	3362263	3362306	3.52E-03
SPPL2B	3816424	3816452	3.52E-03
SLC35B3	2940987	2941009	3.52E-03
TRIO	2802398	2802507	3.52E-03
CRTC1	3825292	3825369	3.53E-03
FCHSD1	2878778	2878792	3.53E-03
ABCA12	2598145	2598185	3.54E-03
AKAP9	3012381	3012499	3.54E-03
TRMT6	3896524	3896539	3.55E-03

SLCO4A1	3892812	3892846	3.55E-03
RASGRF2	2817941	2817998	3.55E-03
SLMAP	2625793	2625843	3.55E-03
ZNF790	3860491	3860536	3.55E-03
SERPINI1	2651165	2651180	3.56E-03
SMEK1	3576545	3576552	3.56E-03
CFTR	3020646	3020699	3.56E-03
GPHN	3540862	3540864	3.56E-03
ESCO1	3800779	3800797	3.56E-03
FASN	3774635	3774687	3.57E-03
LLGL2	3734903	3734926	3.57E-03
PRKCA	3731826	3732006	3.57E-03
CASC1	3447798	3447800	3.58E-03
C2orf65	2560317	2560350	3.58E-03
AGPAT2	3230282	3230287	3.59E-03
TTK	2914777	2914791	3.60E-03
C2orf19	2605660	2605665	3.60E-03
CDCA8	2330773	2330788	3.60E-03
MMP28	3753760	3753784	3.60E-03
ROBO3	3354293	3354340	3.61E-03
C12orf24	3431553	3431556	3.61E-03
HYDIN	3697434	3697486	3.62E-03
PHC3	2704894	2704908	3.62E-03
ZNF550	3872310	3872315	3.62E-03
NT5DC3	3468743	3468752	3.63E-03
UBE2CBP	2962683	2962731	3.63E-03
USF2	3830277	3830279	3.63E-03
B4GALNT3	3400236	3400238	3.64E-03
SLC35B2	2955061	2955068	3.65E-03
EDN2	2408643	2408669	3.66E-03
MTTP	2737257	2737279	3.66E-03
TET1	3249886	3249904	3.66E-03
FLNA	4027176	4027177	3.68E-03
GNB3	3402874	3402880	3.68E-03
	3259367	3259382	3.68E-03
DTWD1	3593452	3593479	3.68E-03
HIP1	3057370	3057400	3.68E-03
MSH5	2902633	2902656	3.69E-03
SMC6	2541944	2541963	3.69E-03
	3874584	3874587	3.69E-03
ZNF493	3826601	3826620	3.69E-03
UTP3	2730531	2730532	3.70E-03
ATP2A1	3655060	3655080	3.70E-03
PDE2A	3381150	3381154	3.71E-03
LRRC6	3154136	3154144	3.71E-03
C8orf41	3130823	3130838	3.71E-03
FAM178A	3260829	3260839	3.71E-03

ERN1	3766651	3766657	3.72E-03
NAE1	3695268	3695269	3.72E-03
SLC12A5	3887241	3887275	3.72E-03
GLYR1	3678395	3678402	3.72E-03
CDH4	3892067	3892091	3.73E-03
ECE2	2655606	2655621	3.73E-03
LHX4	2370032	2370061	3.73E-03
PCDHGA9	2832533	2832624	3.74E-03
VWA3B	2495279	2495400	3.75E-03
SNAPIN	2359764	2359770	3.75E-03
TGDS	3520989	3521001	3.75E-03
USP34	2555277	2555401	3.76E-03
DUS4L	3018509	3018534	3.76E-03
ZBTB2	2979679	2979680	3.76E-03
ATP2A2	3431483	3431539	3.77E-03
EML4	2478748	2478794	3.77E-03
CYP19A1	3624003	3624038	3.77E-03
ATF6B	2949622	2949729	3.78E-03
ESRRB	3545022	3545056	3.78E-03
AMOTL1	3345222	3345248	3.78E-03
ZNF26	3439063	3439109	3.78E-03
YEATS2	2655168	2655248	3.79E-03
NKTR	2619344	2619386	3.79E-03
ZNF425	3078493	3078496	3.79E-03
FASN	3774635	3774663	3.80E-03
FIGNL1	3050367	3050380	3.80E-03
DEPDC4	3467788	3467789	3.80E-03
SEC31B	3303478	3303513	3.81E-03
DTX1	3432556	3432579	3.81E-03
CEP170	2463864	2463865	3.81E-03
C5orf24	2829542	2829549	3.81E-03
RASEF	3211938	3211983	3.82E-03
PAFAH2	2402493	2402509	3.82E-03
WNK2	3179706	3179791	3.82E-03
MLL5	3017547	3017616	3.83E-03
HTR1F	2631940	2631977	3.83E-03
CNKSR1	2326327	2326339	3.84E-03
NIPSNAP1	3956909	3956913	3.84E-03
TEKT4	2493746	2493758	3.84E-03
KRT2	3455692	3455714	3.84E-03
ESCO1	3800779	3800796	3.85E-03
CCDC91	3409432	3409503	3.85E-03
RNASEL	2447124	2447129	3.85E-03
MMP11	3939470	3939477	3.86E-03
C9orf100	3204692	3204712	3.86E-03
TTC37	2867693	2867708	3.86E-03
GABRA2	2767972	2768011	3.88E-03

KPNA6	2328674	2328698	3.88E-03
PI4K2B	2721777	2721800	3.88E-03
ZNF461	3860596	3860606	3.88E-03
RELA	3377789	3377803	3.88E-03
FLJ41603	2835166	2835193	3.89E-03
HERC2	3614901	3614922	3.89E-03
IGSF1	4021777	4021789	3.89E-03
MGAT4A	2566414	2566450	3.89E-03
ABI2	2523689	2523735	3.90E-03
GDAP1	3103607	3103630	3.91E-03
MMP15	3663074	3663090	3.91E-03
SBF1	3965833	3965908	3.91E-03
SCGB2A1	3333417	3333423	3.92E-03
WNT1	3413701	3413715	3.92E-03
FBLIM1	2322036	2322038	3.92E-03
ZNF154	3872380	3872386	3.93E-03
KCNN4	3864646	3864659	3.93E-03
FERMT2	3564919	3564951	3.93E-03
UNC84B	3960827	3960834	3.94E-03
KDR	2769810	2769863	3.94E-03
CHRNA4	3913775	3913789	3.94E-03
ALLC	2467249	2467291	3.95E-03
KIAA0895L	3695450	3695462	3.95E-03
IGFBP5	2598828	2598834	3.96E-03
LIPI	3925381	3925409	3.96E-03
KCTD10	3470793	3470796	3.96E-03
ADAP1	3034987	3034993	3.97E-03
SDCCAG3	3229943	3229974	3.97E-03
RCSD1	2365872	2365921	3.99E-03
	2805482	2805487	3.99E-03
LY96	3103523	3103532	4.00E-03
ADAMTSL3	3605395	3605447	4.00E-03
ALOX12	3708160	3708162	4.01E-03
PIGB	3594825	3594871	4.01E-03
ZNF638	2488114	2488209	4.01E-03
GOLGB1	2691718	2691746	4.02E-03
SLC39A12	3237352	3237364	4.02E-03
ATP13A2	2398736	2398748	4.02E-03
ARHGAP12	3283920	3283968	4.03E-03
C9orf156	3216931	3216951	4.03E-03
CCDC146	3009838	3009885	4.03E-03
ZNF644	2422517	2422527	4.03E-03
EIF2C3	2330133	2330194	4.04E-03
NRAS	2429261	2429263	4.04E-03
NIPSNAP3A	3182957	3182979	4.05E-03
FLJ25328	3823488	3823501	4.07E-03
LRP1	3417842	3417857	4.07E-03

RAD17	2813524	2813540	4.07E-03
CHID1	3358538	3358584	4.08E-03
ZMAT4	3132616	3132649	4.08E-03
CALCR	3061538	3061548	4.09E-03
TRIM31	2948205	2948231	4.10E-03
TCF7L1	2491386	2491396	4.11E-03
PPP2R1B	3391029	3391059	4.11E-03
C1orf63	2402111	2402114	4.11E-03
ATM	3347658	3347686	4.11E-03
SMEK3P	4003500	4003510	4.12E-03
MYH10	3744463	3744569	4.12E-03
CEP135	2727976	2727978	4.13E-03
BSG	3815014	3815021	4.13E-03
SH3YL1	2537109	2537112	4.14E-03
HSPA14	3236395	3236435	4.14E-03
C7orf27	3035795	3035799	4.15E-03
IGSF9	2439861	2439892	4.15E-03
NGEF	2603987	2604014	4.16E-03
CASS4	3890180	3890183	4.16E-03
ZG16B	3645359	3645362	4.16E-03
KIAA0195	3734797	3734860	4.17E-03
C20orf27	3895679	3895687	4.18E-03
ERCC8	2858668	2858674	4.19E-03
	3686635	3686645	4.21E-03
GPR160	2651835	2651870	4.21E-03
CC2D1B	2412740	2412766	4.21E-03
ZNF551	3843463	3843474	4.21E-03
NRK	3986087	3986132	4.22E-03
PIBF1	3493448	3493466	4.22E-03
STK36	2527971	2527974	4.22E-03
RYR1	3832457	3832524	4.22E-03
TNR	2444899	2444912	4.23E-03
SLCO1B3	3407629	3407663	4.24E-03
ZNF83	3869650	3869652	4.25E-03
HPRT1	3991698	3991712	4.25E-03
PIK3R2	3824838	3824841	4.25E-03
TNS3	3049522	3049595	4.26E-03
THOC1	3795680	3795713	4.27E-03
HPCAL1	7385611	2469440	4.28E-03
RBM12	3904119	3904152	4.28E-03
HSD3B1	2354553	2354567	4.28E-03
ACOT12	2864796	2864809	4.28E-03
ERBB3	3417249	3417259	4.28E-03
C6orf227	2950771	2950774	4.28E-03
ATP2B3	3995666	3995692	4.28E-03
ARHGEF15	3709604	3709609	4.29E-03
CXorf40A	3994451	3994459	4.29E-03

ADAP1	3034987	3034992	4.30E-03
GPS1	3738490	3738503	4.30E-03
VWA3B	2495279	2495359	4.31E-03
ADCK1	3545564	3545592	4.31E-03
NEK1	2793221	2793268	4.31E-03
RPE65	2417500	2417513	4.33E-03
COPS8	2534126	2534137	4.33E-03
SPAG5	3750785	3750828	4.33E-03
GPC6	3496637	3496640	4.33E-03
RECQL	3446796	3446811	4.33E-03
SYCP1	2353021	2353066	4.34E-03
ZNHIT3	3719112	3719119	4.34E-03
MRPS27	2861952	2861988	4.34E-03
GALNTL4	3363091	3363108	4.36E-03
RELA	3377789	3377796	4.36E-03
C13orf23	3510362	3510373	4.36E-03
MTHFD1L	2931391	2931484	4.37E-03
ESCO1	3800779	3800795	4.37E-03
RANBP2	2499158	2499188	4.37E-03
LRCH4	3064082	3064107	4.37E-03
CCDC52	2689112	2689130	4.38E-03
ADCY1	3000342	3000416	4.39E-03
MRPL44	2529782	2529784	4.39E-03
TP53RK	3908052	3908056	4.40E-03
QSOX2	3229797	3229798	4.40E-03
OLFM1	3193725	3193743	4.40E-03
C2orf67	2597273	2597288	4.41E-03
OGFOD2	3435653	3435674	4.41E-03
AOAH	3046062	3046135	4.41E-03
TRIM28	3844238	3844255	4.41E-03
OPN1SW	3071860	3071865	4.42E-03
PRKD1	3559192	3559287	4.44E-03
ENOSF1	3795866	3795906	4.44E-03
C16orf67	3657286	3657288	4.45E-03
KIAA0513	3672059	3672071	4.45E-03
SBF1	3965833	3965909	4.45E-03
KIAA1609	3702499	3702500	4.45E-03
TSPYL5	3145801	3145813	4.46E-03
C11orf35	3358112	3358136	4.46E-03
PAPOLG	2484305	2484315	4.46E-03
CBR4	2793054	2793095	4.46E-03
PTPRT	3906390	3906486	4.47E-03
UPP1	3000953	3000954	4.47E-03
PRPF39	3534201	3534233	4.48E-03
CACNB2	3237396	3237462	4.48E-03
ZC3HC1	3072368	3072400	4.48E-03
CDCA4	3581386	3581394	4.48E-03

TRIM54	2474430	2474444	4.48E-03
CREBBP	3677795	3677888	4.48E-03
EZH2	3078348	3078386	4.49E-03
SH3RF1	2793137	2793156	4.50E-03
DDR1	2901970	2901988	4.50E-03
EPS15L1	3853814	3853884	4.50E-03
KIAA0913	3251926	3251950	4.50E-03
C20orf132	3904797	3904821	4.51E-03
SLC35A1	2916345	2916382	4.51E-03
CPB2	3512843	3512847	4.52E-03
RTTN	3812426	3812492	4.52E-03
SLMAP	2625793	2625825	4.52E-03
OVOL1	3335571	3335588	4.52E-03
ATP12A	3481890	3481925	4.52E-03
PQBP1	3976930	3976940	4.53E-03
RTN2	3865422	3865424	4.53E-03
LONP1	3847356	3847371	4.53E-03
LIPF	3256914	3256929	4.53E-03
KCTD15	3829471	3829478	4.53E-03
ARMC9	2531779	2531798	4.54E-03
MARK1	2381177	2381241	4.54E-03
HLX	2381368	2381389	4.55E-03
CNTNAP4	3669171	3669203	4.55E-03
LRDD	3358425	3358435	4.55E-03
CTBS	2420467	2420470	4.55E-03
TLR10	2766192	2766195	4.56E-03
	2769095	2769100	4.56E-03
AHCY	3903361	3903386	4.57E-03
ZMAT1	4016001	4016008	4.58E-03
LOC96610	3938384	3938416	4.58E-03
PCDHB13	2832447	2832457	4.58E-03
CDC40	2921086	2921129	4.59E-03
GTF2I	3008376	3008409	4.59E-03
MAP3K6	2403027	2403038	4.60E-03
DPH5	2425447	2425461	4.61E-03
IQGAP3	2438282	2438333	4.61E-03
HSD3B7	3656737	3656749	4.61E-03
CNTN4	2607923	2608065	4.61E-03
10-Mar	3766013	3766037	4.63E-03
PPP4C	3655961	3655964	4.63E-03
DKFZp451A211	3526495	3526500	4.63E-03
HOOK2	3851720	3851746	4.64E-03
TRIM23	2859734	2859745	4.64E-03
KIAA0746	2764192	2764258	4.65E-03
ELAVL2	3201784	3201816	4.65E-03
FBXL6	3158478	3158483	4.65E-03
PLXNA3	3996467	3996487	4.66E-03

FGFR2	3310041	3310147	4.67E-03
ZNF280D	3625823	3625884	4.67E-03
TAF1D	3386814	3386853	4.68E-03
NOTCH1	3230141	3230180	4.68E-03
HAUS5	3830571	3830583	4.69E-03
SYNGR4	3837744	3837751	4.69E-03
MAOB	4006210	4006224	4.70E-03
CACNA1G	3726618	3726633	4.70E-03
KIAA1586	2911257	2911269	4.71E-03
VPS37B	3475838	3475841	4.71E-03
DHRS3	2397025	2397045	4.71E-03
RYBP	2682436	2682437	4.72E-03
KIF9	2672629	2672666	4.73E-03
CSRP3	3365757	3365767	4.73E-03
HMG20A	3602873	3602904	4.73E-03
SLC2A13	3450899	3450943	4.74E-03
RORC	2435261	2435276	4.74E-03
SLC17A9	3893086	3893103	4.75E-03
GALNT9	3479015	3479027	4.76E-03
NUDCD1	3148796	3148814	4.76E-03
RAB1B	3336074	3336086	4.76E-03
PPP1R15B	2452049	2452050	4.76E-03
JAG1	3897505	3897555	4.76E-03
ABCA8	3768627	3768696	4.77E-03
PRODH2	3860003	3860021	4.77E-03
FAIM	2644702	2644713	4.78E-03
OGDHL	3288803	3288813	4.78E-03
COL11A1	2425756	2425793	4.78E-03
HACL1	2664395	2664416	4.79E-03
HOXA3	3042777	3042788	4.80E-03
USP36	3772581	3772648	4.80E-03
CRH	3138618	3138620	4.81E-03
SCFD1	3531032	3531051	4.81E-03
SPATC1	3119970	3119979	4.82E-03
PON1	3061942	3061943	4.82E-03
SMARCAD1	2736259	2736292	4.83E-03
C6orf204	2971564	2971587	4.83E-03
PDXK	3923257	3923294	4.85E-03
FAM175B	3269280	3269281	4.85E-03
SLC35B3	2940987	2941001	4.85E-03
KDM4B	3817733	3817837	4.85E-03
TTC17	3327948	3327966	4.85E-03
GDF1	3855285	3855291	4.86E-03
SH3YL1	2537109	2537114	4.86E-03
HSPG2	2400793	2400928	4.86E-03
RIPK3	3558226	3558245	4.87E-03
EPHA10	2407314	2407331	4.88E-03

OCRL	3990374	3990390	4.88E-03
SBNO2	3844978	3844981	4.88E-03
LAMA5	3913018	3913022	4.88E-03
ITGA11	3630736	3630804	4.89E-03
AMZ2	3732736	3732780	4.90E-03
C18orf32	3807474	3807475	4.91E-03
FMO1	2367050	2367051	4.92E-03
HOXB3	3761313	3761315	4.92E-03
BAZ1A	3560711	3560713	4.92E-03
C12orf36	3445252	3445261	4.93E-03
C7orf64	3012677	3012682	4.93E-03
RUFY2	3292634	3292668	4.93E-03
AGGF1	2816563	2816585	4.93E-03
MYH14	3839206	3839213	4.94E-03
STAG1	2696802	2696803	4.94E-03
ITGB3BP	2416218	2416245	4.94E-03
C3orf51	2677624	2677626	4.94E-03
HELQ	2776026	2776066	4.95E-03
TM9SF3	3301857	3301862	4.95E-03
PTP4A1	2911903	2911933	4.96E-03
ANKS3	3678279	3678312	4.96E-03
NOTCH3	3853108	3853162	4.96E-03
CDC40	2921086	2921119	4.96E-03
GPR124	3094334	3094338	4.96E-03
GIN1	2869275	2869279	4.96E-03
ITLN2	2440440	2440450	4.97E-03
RBAK	2988459	2988484	4.97E-03
KCNIP4	2762944	2762970	4.97E-03
KCNK10	3575241	3575287	4.97E-03
GLI2	2503374	2503433	4.97E-03
TULP2	3867493	3867498	4.97E-03
C19orf59	3819088	3819090	4.97E-03
CAND1	3420713	3420747	4.99E-03
BUB1B	3589697	3589714	5.00E-03
	2713837	2713854	5.00E-03
MYO1D	3752709	3752793	5.00E-03

Fold-Change (Vasospasm vs. No vasospasm)	Fold-Change (Vasospasm vs. No vasospasm) (Description)
-1.37	Vasospasm down vs No vasospasm
1.74	Vasospasm up vs No vasospasm
-1.35	Vasospasm down vs No vasospasm
-1.52	Vasospasm down vs No vasospasm
-1.54	Vasospasm down vs No vasospasm
-1.57	Vasospasm down vs No vasospasm
-1.46	Vasospasm down vs No vasospasm
1.47	Vasospasm up vs No vasospasm
-1.57	Vasospasm down vs No vasospasm
-1.26	Vasospasm down vs No vasospasm
-1.61	Vasospasm down vs No vasospasm
-1.38	Vasospasm down vs No vasospasm
-1.38	Vasospasm down vs No vasospasm
-1.29	Vasospasm down vs No vasospasm
-1.33	Vasospasm down vs No vasospasm
1.55	Vasospasm up vs No vasospasm
-1.31	Vasospasm down vs No vasospasm
1.22	Vasospasm up vs No vasospasm
-1.38	Vasospasm down vs No vasospasm
-1.32	Vasospasm down vs No vasospasm
-1.45	Vasospasm down vs No vasospasm
1.59	Vasospasm up vs No vasospasm
1.62	Vasospasm up vs No vasospasm
-1.32	Vasospasm down vs No vasospasm
-1.57	Vasospasm down vs No vasospasm
-1.28	Vasospasm down vs No vasospasm
-1.30	Vasospasm down vs No vasospasm
-1.49	Vasospasm down vs No vasospasm
-1.43	Vasospasm down vs No vasospasm
-1.36	Vasospasm down vs No vasospasm
-1.43	Vasospasm down vs No vasospasm
-1.78	Vasospasm down vs No vasospasm
-1.65	Vasospasm down vs No vasospasm
-1.58	Vasospasm down vs No vasospasm
1.63	Vasospasm up vs No vasospasm
-1.25	Vasospasm down vs No vasospasm
-1.41	Vasospasm down vs No vasospasm
-1.70	Vasospasm down vs No vasospasm
-1.42	Vasospasm down vs No vasospasm
1.94	Vasospasm up vs No vasospasm
1.25	Vasospasm up vs No vasospasm
-1.51	Vasospasm down vs No vasospasm
-1.44	Vasospasm down vs No vasospasm
-1.34	Vasospasm down vs No vasospasm
-1.40	Vasospasm down vs No vasospasm
1.55	Vasospasm up vs No vasospasm

-1.47	Vasospasm down vs No vasospasm
-1.44	Vasospasm down vs No vasospasm
-1.30	Vasospasm down vs No vasospasm
-1.41	Vasospasm down vs No vasospasm
2.27	Vasospasm up vs No vasospasm
-1.33	Vasospasm down vs No vasospasm
-1.49	Vasospasm down vs No vasospasm
-1.37	Vasospasm down vs No vasospasm
-1.54	Vasospasm down vs No vasospasm
1.55	Vasospasm up vs No vasospasm
1.99	Vasospasm up vs No vasospasm
-1.31	Vasospasm down vs No vasospasm
1.64	Vasospasm up vs No vasospasm
2.15	Vasospasm up vs No vasospasm
-1.40	Vasospasm down vs No vasospasm
-1.44	Vasospasm down vs No vasospasm
-1.63	Vasospasm down vs No vasospasm
-1.30	Vasospasm down vs No vasospasm
-1.59	Vasospasm down vs No vasospasm
-1.71	Vasospasm down vs No vasospasm
-1.41	Vasospasm down vs No vasospasm
-1.47	Vasospasm down vs No vasospasm
1.58	Vasospasm up vs No vasospasm
-1.25	Vasospasm down vs No vasospasm
1.28	Vasospasm up vs No vasospasm
-1.40	Vasospasm down vs No vasospasm
1.93	Vasospasm up vs No vasospasm
1.91	Vasospasm up vs No vasospasm
-1.73	Vasospasm down vs No vasospasm
-1.36	Vasospasm down vs No vasospasm
-1.44	Vasospasm down vs No vasospasm
1.77	Vasospasm up vs No vasospasm
-1.53	Vasospasm down vs No vasospasm
1.46	Vasospasm up vs No vasospasm
-1.49	Vasospasm down vs No vasospasm
1.64	Vasospasm up vs No vasospasm
2.01	Vasospasm up vs No vasospasm
-1.35	Vasospasm down vs No vasospasm
-1.52	Vasospasm down vs No vasospasm
-1.21	Vasospasm down vs No vasospasm
-1.80	Vasospasm down vs No vasospasm
-1.62	Vasospasm down vs No vasospasm
1.92	Vasospasm up vs No vasospasm
-1.60	Vasospasm down vs No vasospasm
-1.46	Vasospasm down vs No vasospasm
1.31	Vasospasm up vs No vasospasm
1.61	Vasospasm up vs No vasospasm

1.67	Vasospasm up vs No vasospasm
-1.39	Vasospasm down vs No vasospasm
-1.52	Vasospasm down vs No vasospasm
2.55	Vasospasm up vs No vasospasm
1.35	Vasospasm up vs No vasospasm
-1.24	Vasospasm down vs No vasospasm
1.48	Vasospasm up vs No vasospasm
-1.47	Vasospasm down vs No vasospasm
-1.61	Vasospasm down vs No vasospasm
-1.45	Vasospasm down vs No vasospasm
1.65	Vasospasm up vs No vasospasm
-1.24	Vasospasm down vs No vasospasm
-1.27	Vasospasm down vs No vasospasm
-1.24	Vasospasm down vs No vasospasm
-1.33	Vasospasm down vs No vasospasm
-1.23	Vasospasm down vs No vasospasm
-1.59	Vasospasm down vs No vasospasm
-1.44	Vasospasm down vs No vasospasm
-1.33	Vasospasm down vs No vasospasm
-1.23	Vasospasm down vs No vasospasm
1.48	Vasospasm up vs No vasospasm
-1.59	Vasospasm down vs No vasospasm
1.44	Vasospasm up vs No vasospasm
-1.42	Vasospasm down vs No vasospasm
2.35	Vasospasm up vs No vasospasm
-1.39	Vasospasm down vs No vasospasm
-1.31	Vasospasm down vs No vasospasm
1.47	Vasospasm up vs No vasospasm
-1.31	Vasospasm down vs No vasospasm
1.58	Vasospasm up vs No vasospasm
-1.46	Vasospasm down vs No vasospasm
1.52	Vasospasm up vs No vasospasm
-1.47	Vasospasm down vs No vasospasm
-1.24	Vasospasm down vs No vasospasm
-1.37	Vasospasm down vs No vasospasm
-1.34	Vasospasm down vs No vasospasm
2.00	Vasospasm up vs No vasospasm
1.85	Vasospasm up vs No vasospasm
-1.26	Vasospasm down vs No vasospasm
-1.35	Vasospasm down vs No vasospasm
-1.39	Vasospasm down vs No vasospasm
-1.22	Vasospasm down vs No vasospasm
1.67	Vasospasm up vs No vasospasm
-1.26	Vasospasm down vs No vasospasm
-1.46	Vasospasm down vs No vasospasm
-1.37	Vasospasm down vs No vasospasm
-1.57	Vasospasm down vs No vasospasm

2.14	Vasospasm up vs No vasospasm
-1.42	Vasospasm down vs No vasospasm
-1.42	Vasospasm down vs No vasospasm
2.08	Vasospasm up vs No vasospasm
1.51	Vasospasm up vs No vasospasm
1.56	Vasospasm up vs No vasospasm
1.54	Vasospasm up vs No vasospasm
-1.32	Vasospasm down vs No vasospasm
-1.26	Vasospasm down vs No vasospasm
-1.31	Vasospasm down vs No vasospasm
-1.58	Vasospasm down vs No vasospasm
-1.45	Vasospasm down vs No vasospasm
1.74	Vasospasm up vs No vasospasm
1.78	Vasospasm up vs No vasospasm
1.89	Vasospasm up vs No vasospasm
1.47	Vasospasm up vs No vasospasm
1.58	Vasospasm up vs No vasospasm
1.79	Vasospasm up vs No vasospasm
2.00	Vasospasm up vs No vasospasm
-1.27	Vasospasm down vs No vasospasm
-1.28	Vasospasm down vs No vasospasm
-1.23	Vasospasm down vs No vasospasm
-1.46	Vasospasm down vs No vasospasm
-1.45	Vasospasm down vs No vasospasm
-1.55	Vasospasm down vs No vasospasm
-1.59	Vasospasm down vs No vasospasm
1.21	Vasospasm up vs No vasospasm
-1.35	Vasospasm down vs No vasospasm
-1.33	Vasospasm down vs No vasospasm
-1.26	Vasospasm down vs No vasospasm
1.34	Vasospasm up vs No vasospasm
1.53	Vasospasm up vs No vasospasm
-1.71	Vasospasm down vs No vasospasm
-1.68	Vasospasm down vs No vasospasm
1.58	Vasospasm up vs No vasospasm
-1.22	Vasospasm down vs No vasospasm
-1.30	Vasospasm down vs No vasospasm
-1.50	Vasospasm down vs No vasospasm
2.06	Vasospasm up vs No vasospasm
-1.51	Vasospasm down vs No vasospasm
-1.22	Vasospasm down vs No vasospasm
-1.29	Vasospasm down vs No vasospasm
1.63	Vasospasm up vs No vasospasm
1.30	Vasospasm up vs No vasospasm
1.59	Vasospasm up vs No vasospasm
-1.51	Vasospasm down vs No vasospasm
-1.60	Vasospasm down vs No vasospasm

1.57	Vasospasm up vs No vasospasm
-1.29	Vasospasm down vs No vasospasm
-1.28	Vasospasm down vs No vasospasm
-1.36	Vasospasm down vs No vasospasm
-1.57	Vasospasm down vs No vasospasm
1.70	Vasospasm up vs No vasospasm
-1.48	Vasospasm down vs No vasospasm
-1.24	Vasospasm down vs No vasospasm
1.25	Vasospasm up vs No vasospasm
-1.38	Vasospasm down vs No vasospasm
1.80	Vasospasm up vs No vasospasm
-1.27	Vasospasm down vs No vasospasm
-1.27	Vasospasm down vs No vasospasm
-1.37	Vasospasm down vs No vasospasm
1.44	Vasospasm up vs No vasospasm
-1.35	Vasospasm down vs No vasospasm
-1.32	Vasospasm down vs No vasospasm
-1.31	Vasospasm down vs No vasospasm
-1.22	Vasospasm down vs No vasospasm
1.69	Vasospasm up vs No vasospasm
-1.48	Vasospasm down vs No vasospasm
-1.23	Vasospasm down vs No vasospasm
1.38	Vasospasm up vs No vasospasm
-1.23	Vasospasm down vs No vasospasm
-1.26	Vasospasm down vs No vasospasm
1.64	Vasospasm up vs No vasospasm
-1.38	Vasospasm down vs No vasospasm
1.72	Vasospasm up vs No vasospasm
1.50	Vasospasm up vs No vasospasm
1.42	Vasospasm up vs No vasospasm
-1.26	Vasospasm down vs No vasospasm
1.32	Vasospasm up vs No vasospasm
-1.48	Vasospasm down vs No vasospasm
2.29	Vasospasm up vs No vasospasm
1.50	Vasospasm up vs No vasospasm
1.50	Vasospasm up vs No vasospasm
-1.25	Vasospasm down vs No vasospasm
-1.32	Vasospasm down vs No vasospasm
1.22	Vasospasm up vs No vasospasm
-1.41	Vasospasm down vs No vasospasm
1.80	Vasospasm up vs No vasospasm
-1.20	Vasospasm down vs No vasospasm
-1.41	Vasospasm down vs No vasospasm
-1.37	Vasospasm down vs No vasospasm
-1.26	Vasospasm down vs No vasospasm
1.33	Vasospasm up vs No vasospasm
-1.26	Vasospasm down vs No vasospasm

-1.30	Vasospasm down vs No vasospasm
-1.41	Vasospasm down vs No vasospasm
-1.57	Vasospasm down vs No vasospasm
-1.26	Vasospasm down vs No vasospasm
-1.45	Vasospasm down vs No vasospasm
-1.31	Vasospasm down vs No vasospasm
1.68	Vasospasm up vs No vasospasm
-1.44	Vasospasm down vs No vasospasm
1.39	Vasospasm up vs No vasospasm
-1.26	Vasospasm down vs No vasospasm
1.35	Vasospasm up vs No vasospasm
-1.26	Vasospasm down vs No vasospasm
-1.30	Vasospasm down vs No vasospasm
-1.34	Vasospasm down vs No vasospasm
-1.35	Vasospasm down vs No vasospasm
-1.42	Vasospasm down vs No vasospasm
-1.42	Vasospasm down vs No vasospasm
-1.62	Vasospasm down vs No vasospasm
1.40	Vasospasm up vs No vasospasm
-1.49	Vasospasm down vs No vasospasm
-1.34	Vasospasm down vs No vasospasm
-1.47	Vasospasm down vs No vasospasm
1.40	Vasospasm up vs No vasospasm
-1.28	Vasospasm down vs No vasospasm
1.48	Vasospasm up vs No vasospasm
-1.45	Vasospasm down vs No vasospasm
-1.48	Vasospasm down vs No vasospasm
-1.76	Vasospasm down vs No vasospasm
1.39	Vasospasm up vs No vasospasm
-1.65	Vasospasm down vs No vasospasm
-1.38	Vasospasm down vs No vasospasm
1.53	Vasospasm up vs No vasospasm
1.51	Vasospasm up vs No vasospasm
1.55	Vasospasm up vs No vasospasm
-1.57	Vasospasm down vs No vasospasm
1.66	Vasospasm up vs No vasospasm
-1.34	Vasospasm down vs No vasospasm
-1.48	Vasospasm down vs No vasospasm
-1.25	Vasospasm down vs No vasospasm
-1.29	Vasospasm down vs No vasospasm
-1.22	Vasospasm down vs No vasospasm
-1.32	Vasospasm down vs No vasospasm
1.95	Vasospasm up vs No vasospasm
-1.32	Vasospasm down vs No vasospasm
-1.66	Vasospasm down vs No vasospasm
1.62	Vasospasm up vs No vasospasm
-1.25	Vasospasm down vs No vasospasm

-1.31	Vasospasm down vs No vasospasm
-1.26	Vasospasm down vs No vasospasm
-1.41	Vasospasm down vs No vasospasm
-1.29	Vasospasm down vs No vasospasm
-1.20	Vasospasm down vs No vasospasm
1.39	Vasospasm up vs No vasospasm
-1.56	Vasospasm down vs No vasospasm
-1.40	Vasospasm down vs No vasospasm
-1.26	Vasospasm down vs No vasospasm
-1.24	Vasospasm down vs No vasospasm
-1.58	Vasospasm down vs No vasospasm
-1.50	Vasospasm down vs No vasospasm
-1.39	Vasospasm down vs No vasospasm
1.55	Vasospasm up vs No vasospasm
-1.42	Vasospasm down vs No vasospasm
1.39	Vasospasm up vs No vasospasm
-1.34	Vasospasm down vs No vasospasm
-2.10	Vasospasm down vs No vasospasm
-1.41	Vasospasm down vs No vasospasm
-1.38	Vasospasm down vs No vasospasm
-1.42	Vasospasm down vs No vasospasm
-1.37	Vasospasm down vs No vasospasm
1.56	Vasospasm up vs No vasospasm
1.77	Vasospasm up vs No vasospasm
1.51	Vasospasm up vs No vasospasm
1.34	Vasospasm up vs No vasospasm
-1.38	Vasospasm down vs No vasospasm
-1.44	Vasospasm down vs No vasospasm
-1.32	Vasospasm down vs No vasospasm
-1.27	Vasospasm down vs No vasospasm
1.71	Vasospasm up vs No vasospasm
-1.23	Vasospasm down vs No vasospasm
-1.24	Vasospasm down vs No vasospasm
1.36	Vasospasm up vs No vasospasm
-1.55	Vasospasm down vs No vasospasm
1.32	Vasospasm up vs No vasospasm
-1.29	Vasospasm down vs No vasospasm
-1.30	Vasospasm down vs No vasospasm
1.36	Vasospasm up vs No vasospasm
1.45	Vasospasm up vs No vasospasm
1.45	Vasospasm up vs No vasospasm
-1.49	Vasospasm down vs No vasospasm
-1.43	Vasospasm down vs No vasospasm
1.30	Vasospasm up vs No vasospasm
-1.45	Vasospasm down vs No vasospasm
-1.24	Vasospasm down vs No vasospasm
-1.43	Vasospasm down vs No vasospasm

-1.26	Vasospasm down vs No vasospasm
1.59	Vasospasm up vs No vasospasm
-1.58	Vasospasm down vs No vasospasm
1.22	Vasospasm up vs No vasospasm
-1.47	Vasospasm down vs No vasospasm
-1.69	Vasospasm down vs No vasospasm
1.54	Vasospasm up vs No vasospasm
-1.62	Vasospasm down vs No vasospasm
1.31	Vasospasm up vs No vasospasm
-1.37	Vasospasm down vs No vasospasm
2.22	Vasospasm up vs No vasospasm
-1.35	Vasospasm down vs No vasospasm
-1.26	Vasospasm down vs No vasospasm
-1.31	Vasospasm down vs No vasospasm
-1.35	Vasospasm down vs No vasospasm
-1.20	Vasospasm down vs No vasospasm
-1.51	Vasospasm down vs No vasospasm
-1.82	Vasospasm down vs No vasospasm
1.25	Vasospasm up vs No vasospasm
1.42	Vasospasm up vs No vasospasm
-1.26	Vasospasm down vs No vasospasm
-1.38	Vasospasm down vs No vasospasm
2.20	Vasospasm up vs No vasospasm
-1.42	Vasospasm down vs No vasospasm
-1.49	Vasospasm down vs No vasospasm
-1.45	Vasospasm down vs No vasospasm
1.39	Vasospasm up vs No vasospasm
-1.22	Vasospasm down vs No vasospasm
-1.54	Vasospasm down vs No vasospasm
1.86	Vasospasm up vs No vasospasm
1.71	Vasospasm up vs No vasospasm
-1.47	Vasospasm down vs No vasospasm
-1.58	Vasospasm down vs No vasospasm
-1.63	Vasospasm down vs No vasospasm
-1.33	Vasospasm down vs No vasospasm
-1.22	Vasospasm down vs No vasospasm
-1.58	Vasospasm down vs No vasospasm
-1.61	Vasospasm down vs No vasospasm
-1.24	Vasospasm down vs No vasospasm
1.40	Vasospasm up vs No vasospasm
1.28	Vasospasm up vs No vasospasm
1.99	Vasospasm up vs No vasospasm
-1.37	Vasospasm down vs No vasospasm
1.34	Vasospasm up vs No vasospasm
-1.30	Vasospasm down vs No vasospasm
-1.29	Vasospasm down vs No vasospasm
1.20	Vasospasm up vs No vasospasm

1.27	Vasospasm up vs No vasospasm
-1.22	Vasospasm down vs No vasospasm
-1.80	Vasospasm down vs No vasospasm
-1.65	Vasospasm down vs No vasospasm
1.30	Vasospasm up vs No vasospasm
-1.35	Vasospasm down vs No vasospasm
-1.26	Vasospasm down vs No vasospasm
-1.33	Vasospasm down vs No vasospasm
1.45	Vasospasm up vs No vasospasm
-1.47	Vasospasm down vs No vasospasm
-1.35	Vasospasm down vs No vasospasm
1.25	Vasospasm up vs No vasospasm
-1.35	Vasospasm down vs No vasospasm
-1.37	Vasospasm down vs No vasospasm
1.36	Vasospasm up vs No vasospasm
-1.23	Vasospasm down vs No vasospasm
-1.91	Vasospasm down vs No vasospasm
1.42	Vasospasm up vs No vasospasm
2.29	Vasospasm up vs No vasospasm
-1.33	Vasospasm down vs No vasospasm
-1.24	Vasospasm down vs No vasospasm
-1.23	Vasospasm down vs No vasospasm
1.70	Vasospasm up vs No vasospasm
-1.35	Vasospasm down vs No vasospasm
-1.38	Vasospasm down vs No vasospasm
-1.25	Vasospasm down vs No vasospasm
-1.29	Vasospasm down vs No vasospasm
-1.57	Vasospasm down vs No vasospasm
1.72	Vasospasm up vs No vasospasm
-1.38	Vasospasm down vs No vasospasm
-1.44	Vasospasm down vs No vasospasm
-1.34	Vasospasm down vs No vasospasm
1.42	Vasospasm up vs No vasospasm
1.74	Vasospasm up vs No vasospasm
-1.33	Vasospasm down vs No vasospasm
-1.33	Vasospasm down vs No vasospasm
1.53	Vasospasm up vs No vasospasm
-1.51	Vasospasm down vs No vasospasm
-1.39	Vasospasm down vs No vasospasm
-1.34	Vasospasm down vs No vasospasm
-1.31	Vasospasm down vs No vasospasm
-1.46	Vasospasm down vs No vasospasm
1.55	Vasospasm up vs No vasospasm
-1.57	Vasospasm down vs No vasospasm
1.30	Vasospasm up vs No vasospasm
-1.41	Vasospasm down vs No vasospasm
-1.58	Vasospasm down vs No vasospasm

-1.54	Vasospasm down vs No vasospasm
-1.44	Vasospasm down vs No vasospasm
-1.30	Vasospasm down vs No vasospasm
-1.42	Vasospasm down vs No vasospasm
-1.52	Vasospasm down vs No vasospasm
1.59	Vasospasm up vs No vasospasm
-1.38	Vasospasm down vs No vasospasm
1.53	Vasospasm up vs No vasospasm
-1.66	Vasospasm down vs No vasospasm
1.29	Vasospasm up vs No vasospasm
-1.49	Vasospasm down vs No vasospasm
-1.51	Vasospasm down vs No vasospasm
1.97	Vasospasm up vs No vasospasm
-1.40	Vasospasm down vs No vasospasm
-1.84	Vasospasm down vs No vasospasm
-1.65	Vasospasm down vs No vasospasm
-1.28	Vasospasm down vs No vasospasm
1.62	Vasospasm up vs No vasospasm
-1.41	Vasospasm down vs No vasospasm
-1.27	Vasospasm down vs No vasospasm
1.39	Vasospasm up vs No vasospasm
-1.56	Vasospasm down vs No vasospasm
-1.22	Vasospasm down vs No vasospasm
1.36	Vasospasm up vs No vasospasm
-1.45	Vasospasm down vs No vasospasm
-1.27	Vasospasm down vs No vasospasm
-1.47	Vasospasm down vs No vasospasm
1.37	Vasospasm up vs No vasospasm
-1.33	Vasospasm down vs No vasospasm
-1.21	Vasospasm down vs No vasospasm
-1.29	Vasospasm down vs No vasospasm
-1.28	Vasospasm down vs No vasospasm
1.39	Vasospasm up vs No vasospasm
-1.35	Vasospasm down vs No vasospasm
-1.30	Vasospasm down vs No vasospasm
1.48	Vasospasm up vs No vasospasm
-1.38	Vasospasm down vs No vasospasm
1.47	Vasospasm up vs No vasospasm
-1.42	Vasospasm down vs No vasospasm
-1.41	Vasospasm down vs No vasospasm
-1.21	Vasospasm down vs No vasospasm
-1.48	Vasospasm down vs No vasospasm
1.49	Vasospasm up vs No vasospasm
1.29	Vasospasm up vs No vasospasm
1.41	Vasospasm up vs No vasospasm
1.36	Vasospasm up vs No vasospasm
-1.39	Vasospasm down vs No vasospasm

-1.52	Vasospasm down vs No vasospasm
-1.45	Vasospasm down vs No vasospasm
1.68	Vasospasm up vs No vasospasm
-1.38	Vasospasm down vs No vasospasm
-1.39	Vasospasm down vs No vasospasm
-1.33	Vasospasm down vs No vasospasm
-1.29	Vasospasm down vs No vasospasm
-1.45	Vasospasm down vs No vasospasm
1.32	Vasospasm up vs No vasospasm
-1.42	Vasospasm down vs No vasospasm
-1.30	Vasospasm down vs No vasospasm
-1.40	Vasospasm down vs No vasospasm
-1.38	Vasospasm down vs No vasospasm
1.35	Vasospasm up vs No vasospasm
1.25	Vasospasm up vs No vasospasm
-1.37	Vasospasm down vs No vasospasm
1.73	Vasospasm up vs No vasospasm
-1.31	Vasospasm down vs No vasospasm
1.40	Vasospasm up vs No vasospasm
-1.44	Vasospasm down vs No vasospasm
-1.36	Vasospasm down vs No vasospasm
-1.29	Vasospasm down vs No vasospasm
-1.36	Vasospasm down vs No vasospasm
-1.38	Vasospasm down vs No vasospasm
1.32	Vasospasm up vs No vasospasm
-1.23	Vasospasm down vs No vasospasm
-1.43	Vasospasm down vs No vasospasm
-1.49	Vasospasm down vs No vasospasm
-1.52	Vasospasm down vs No vasospasm
1.25	Vasospasm up vs No vasospasm
-1.35	Vasospasm down vs No vasospasm
-1.52	Vasospasm down vs No vasospasm
-2.23	Vasospasm down vs No vasospasm
-1.33	Vasospasm down vs No vasospasm
-1.26	Vasospasm down vs No vasospasm
1.61	Vasospasm up vs No vasospasm
-1.32	Vasospasm down vs No vasospasm
-1.37	Vasospasm down vs No vasospasm
-1.22	Vasospasm down vs No vasospasm
-1.45	Vasospasm down vs No vasospasm
-1.50	Vasospasm down vs No vasospasm
1.29	Vasospasm up vs No vasospasm
1.40	Vasospasm up vs No vasospasm
1.51	Vasospasm up vs No vasospasm
1.58	Vasospasm up vs No vasospasm
-1.34	Vasospasm down vs No vasospasm
1.29	Vasospasm up vs No vasospasm

-1.37	Vasospasm down vs No vasospasm
1.48	Vasospasm up vs No vasospasm
1.60	Vasospasm up vs No vasospasm
-1.24	Vasospasm down vs No vasospasm
-1.25	Vasospasm down vs No vasospasm
1.56	Vasospasm up vs No vasospasm
-1.28	Vasospasm down vs No vasospasm
2.25	Vasospasm up vs No vasospasm
-1.29	Vasospasm down vs No vasospasm
-1.39	Vasospasm down vs No vasospasm
-1.47	Vasospasm down vs No vasospasm
2.02	Vasospasm up vs No vasospasm
1.84	Vasospasm up vs No vasospasm
-1.44	Vasospasm down vs No vasospasm
-1.52	Vasospasm down vs No vasospasm
-1.47	Vasospasm down vs No vasospasm
1.36	Vasospasm up vs No vasospasm
-1.26	Vasospasm down vs No vasospasm
1.32	Vasospasm up vs No vasospasm
-1.37	Vasospasm down vs No vasospasm
1.59	Vasospasm up vs No vasospasm
-1.41	Vasospasm down vs No vasospasm
-1.23	Vasospasm down vs No vasospasm
-1.27	Vasospasm down vs No vasospasm
-1.44	Vasospasm down vs No vasospasm
-1.39	Vasospasm down vs No vasospasm
1.42	Vasospasm up vs No vasospasm
-1.65	Vasospasm down vs No vasospasm
1.45	Vasospasm up vs No vasospasm
-1.43	Vasospasm down vs No vasospasm
-1.22	Vasospasm down vs No vasospasm
-1.21	Vasospasm down vs No vasospasm
-1.51	Vasospasm down vs No vasospasm
-1.26	Vasospasm down vs No vasospasm
1.88	Vasospasm up vs No vasospasm
-1.43	Vasospasm down vs No vasospasm
1.47	Vasospasm up vs No vasospasm
1.57	Vasospasm up vs No vasospasm
-1.51	Vasospasm down vs No vasospasm
-1.44	Vasospasm down vs No vasospasm
-1.39	Vasospasm down vs No vasospasm
-1.26	Vasospasm down vs No vasospasm
-1.39	Vasospasm down vs No vasospasm
-1.27	Vasospasm down vs No vasospasm
1.22	Vasospasm up vs No vasospasm
1.45	Vasospasm up vs No vasospasm
-1.21	Vasospasm down vs No vasospasm

-1.44	Vasospasm down vs No vasospasm
1.64	Vasospasm up vs No vasospasm
-1.35	Vasospasm down vs No vasospasm
-1.31	Vasospasm down vs No vasospasm
-1.49	Vasospasm down vs No vasospasm
1.60	Vasospasm up vs No vasospasm
-1.39	Vasospasm down vs No vasospasm
-1.53	Vasospasm down vs No vasospasm
-1.57	Vasospasm down vs No vasospasm
-1.23	Vasospasm down vs No vasospasm
-1.41	Vasospasm down vs No vasospasm
-1.39	Vasospasm down vs No vasospasm
-1.46	Vasospasm down vs No vasospasm
-1.27	Vasospasm down vs No vasospasm
1.28	Vasospasm up vs No vasospasm
1.30	Vasospasm up vs No vasospasm
1.25	Vasospasm up vs No vasospasm
1.66	Vasospasm up vs No vasospasm
1.40	Vasospasm up vs No vasospasm
1.37	Vasospasm up vs No vasospasm
-1.26	Vasospasm down vs No vasospasm
-1.62	Vasospasm down vs No vasospasm
-1.36	Vasospasm down vs No vasospasm
-1.21	Vasospasm down vs No vasospasm
-1.45	Vasospasm down vs No vasospasm
1.75	Vasospasm up vs No vasospasm
-1.35	Vasospasm down vs No vasospasm
-1.41	Vasospasm down vs No vasospasm
1.68	Vasospasm up vs No vasospasm
-1.45	Vasospasm down vs No vasospasm
-1.22	Vasospasm down vs No vasospasm
-1.35	Vasospasm down vs No vasospasm
-1.28	Vasospasm down vs No vasospasm
-1.50	Vasospasm down vs No vasospasm
-1.34	Vasospasm down vs No vasospasm
-1.30	Vasospasm down vs No vasospasm
1.33	Vasospasm up vs No vasospasm
1.63	Vasospasm up vs No vasospasm
-1.20	Vasospasm down vs No vasospasm
1.54	Vasospasm up vs No vasospasm
1.87	Vasospasm up vs No vasospasm
-1.32	Vasospasm down vs No vasospasm
-1.34	Vasospasm down vs No vasospasm
-1.29	Vasospasm down vs No vasospasm
1.61	Vasospasm up vs No vasospasm
1.50	Vasospasm up vs No vasospasm
1.54	Vasospasm up vs No vasospasm

-1.34	Vasospasm down vs No vasospasm
-1.23	Vasospasm down vs No vasospasm
-1.40	Vasospasm down vs No vasospasm
1.42	Vasospasm up vs No vasospasm
-1.38	Vasospasm down vs No vasospasm
-1.31	Vasospasm down vs No vasospasm
-1.33	Vasospasm down vs No vasospasm
-1.21	Vasospasm down vs No vasospasm
1.48	Vasospasm up vs No vasospasm
-1.39	Vasospasm down vs No vasospasm
-1.25	Vasospasm down vs No vasospasm
-1.23	Vasospasm down vs No vasospasm
-1.32	Vasospasm down vs No vasospasm
1.53	Vasospasm up vs No vasospasm
1.43	Vasospasm up vs No vasospasm
1.66	Vasospasm up vs No vasospasm
-1.21	Vasospasm down vs No vasospasm
-1.24	Vasospasm down vs No vasospasm
-1.26	Vasospasm down vs No vasospasm
1.44	Vasospasm up vs No vasospasm
-1.56	Vasospasm down vs No vasospasm
-1.33	Vasospasm down vs No vasospasm
1.67	Vasospasm up vs No vasospasm
1.73	Vasospasm up vs No vasospasm
1.76	Vasospasm up vs No vasospasm
-1.21	Vasospasm down vs No vasospasm
-1.26	Vasospasm down vs No vasospasm
-1.22	Vasospasm down vs No vasospasm
-1.41	Vasospasm down vs No vasospasm
-1.35	Vasospasm down vs No vasospasm
-1.33	Vasospasm down vs No vasospasm
-1.29	Vasospasm down vs No vasospasm
1.51	Vasospasm up vs No vasospasm
-1.37	Vasospasm down vs No vasospasm
-1.30	Vasospasm down vs No vasospasm
-1.40	Vasospasm down vs No vasospasm
-1.39	Vasospasm down vs No vasospasm
1.25	Vasospasm up vs No vasospasm
-1.35	Vasospasm down vs No vasospasm
1.64	Vasospasm up vs No vasospasm
-1.43	Vasospasm down vs No vasospasm
1.67	Vasospasm up vs No vasospasm
1.25	Vasospasm up vs No vasospasm
1.25	Vasospasm up vs No vasospasm
1.42	Vasospasm up vs No vasospasm
1.65	Vasospasm up vs No vasospasm
1.96	Vasospasm up vs No vasospasm

-1.23	Vasospasm down vs No vasospasm
1.58	Vasospasm up vs No vasospasm
-1.21	Vasospasm down vs No vasospasm
-1.39	Vasospasm down vs No vasospasm
-1.48	Vasospasm down vs No vasospasm
1.29	Vasospasm up vs No vasospasm
-1.20	Vasospasm down vs No vasospasm
-1.47	Vasospasm down vs No vasospasm
1.75	Vasospasm up vs No vasospasm
-1.23	Vasospasm down vs No vasospasm
1.26	Vasospasm up vs No vasospasm
-1.50	Vasospasm down vs No vasospasm
1.45	Vasospasm up vs No vasospasm
-1.31	Vasospasm down vs No vasospasm
1.54	Vasospasm up vs No vasospasm
-1.30	Vasospasm down vs No vasospasm
-1.40	Vasospasm down vs No vasospasm
-1.36	Vasospasm down vs No vasospasm
-1.31	Vasospasm down vs No vasospasm
-1.26	Vasospasm down vs No vasospasm
1.67	Vasospasm up vs No vasospasm
-1.38	Vasospasm down vs No vasospasm
-1.29	Vasospasm down vs No vasospasm
-1.28	Vasospasm down vs No vasospasm
-1.40	Vasospasm down vs No vasospasm
1.63	Vasospasm up vs No vasospasm
1.33	Vasospasm up vs No vasospasm
-1.48	Vasospasm down vs No vasospasm
1.86	Vasospasm up vs No vasospasm
1.34	Vasospasm up vs No vasospasm
-1.37	Vasospasm down vs No vasospasm
1.68	Vasospasm up vs No vasospasm
-1.32	Vasospasm down vs No vasospasm
-1.35	Vasospasm down vs No vasospasm
-1.23	Vasospasm down vs No vasospasm
-1.31	Vasospasm down vs No vasospasm
-1.21	Vasospasm down vs No vasospasm
-1.23	Vasospasm down vs No vasospasm
-1.40	Vasospasm down vs No vasospasm
-1.55	Vasospasm down vs No vasospasm
-1.40	Vasospasm down vs No vasospasm
1.85	Vasospasm up vs No vasospasm
1.55	Vasospasm up vs No vasospasm
1.65	Vasospasm up vs No vasospasm
1.66	Vasospasm up vs No vasospasm
-1.37	Vasospasm down vs No vasospasm
1.63	Vasospasm up vs No vasospasm

-1.36	Vasospasm down vs No vasospasm
1.47	Vasospasm up vs No vasospasm
-1.34	Vasospasm down vs No vasospasm
-1.34	Vasospasm down vs No vasospasm
1.64	Vasospasm up vs No vasospasm
1.50	Vasospasm up vs No vasospasm
-1.24	Vasospasm down vs No vasospasm
-1.42	Vasospasm down vs No vasospasm
-1.34	Vasospasm down vs No vasospasm
-1.21	Vasospasm down vs No vasospasm
-1.44	Vasospasm down vs No vasospasm
1.28	Vasospasm up vs No vasospasm
1.61	Vasospasm up vs No vasospasm
1.39	Vasospasm up vs No vasospasm
-1.37	Vasospasm down vs No vasospasm
1.28	Vasospasm up vs No vasospasm
-1.20	Vasospasm down vs No vasospasm
-1.34	Vasospasm down vs No vasospasm
-1.24	Vasospasm down vs No vasospasm
-1.23	Vasospasm down vs No vasospasm
1.60	Vasospasm up vs No vasospasm
-1.45	Vasospasm down vs No vasospasm
1.53	Vasospasm up vs No vasospasm
-1.31	Vasospasm down vs No vasospasm
-1.40	Vasospasm down vs No vasospasm
-1.59	Vasospasm down vs No vasospasm
-1.26	Vasospasm down vs No vasospasm
1.35	Vasospasm up vs No vasospasm
-1.28	Vasospasm down vs No vasospasm
1.42	Vasospasm up vs No vasospasm
-1.33	Vasospasm down vs No vasospasm
1.41	Vasospasm up vs No vasospasm
-1.36	Vasospasm down vs No vasospasm
1.31	Vasospasm up vs No vasospasm
-1.40	Vasospasm down vs No vasospasm
-1.26	Vasospasm down vs No vasospasm
1.31	Vasospasm up vs No vasospasm
1.41	Vasospasm up vs No vasospasm
-1.35	Vasospasm down vs No vasospasm
1.56	Vasospasm up vs No vasospasm
-1.28	Vasospasm down vs No vasospasm
-1.28	Vasospasm down vs No vasospasm
-1.33	Vasospasm down vs No vasospasm
1.48	Vasospasm up vs No vasospasm
1.35	Vasospasm up vs No vasospasm
1.79	Vasospasm up vs No vasospasm
-1.28	Vasospasm down vs No vasospasm

-1.62	Vasospasm down vs No vasospasm
-1.27	Vasospasm down vs No vasospasm
-1.25	Vasospasm down vs No vasospasm
1.43	Vasospasm up vs No vasospasm
-1.32	Vasospasm down vs No vasospasm
-1.22	Vasospasm down vs No vasospasm
-1.36	Vasospasm down vs No vasospasm
-1.28	Vasospasm down vs No vasospasm
1.54	Vasospasm up vs No vasospasm
-1.24	Vasospasm down vs No vasospasm
-1.35	Vasospasm down vs No vasospasm
-1.27	Vasospasm down vs No vasospasm
1.70	Vasospasm up vs No vasospasm
1.37	Vasospasm up vs No vasospasm
-1.22	Vasospasm down vs No vasospasm
-1.23	Vasospasm down vs No vasospasm
1.65	Vasospasm up vs No vasospasm
-1.31	Vasospasm down vs No vasospasm
-1.25	Vasospasm down vs No vasospasm
1.44	Vasospasm up vs No vasospasm
-1.57	Vasospasm down vs No vasospasm
-1.24	Vasospasm down vs No vasospasm
-1.21	Vasospasm down vs No vasospasm
1.36	Vasospasm up vs No vasospasm
1.23	Vasospasm up vs No vasospasm
1.57	Vasospasm up vs No vasospasm
-1.26	Vasospasm down vs No vasospasm
1.38	Vasospasm up vs No vasospasm
-1.44	Vasospasm down vs No vasospasm
-1.21	Vasospasm down vs No vasospasm
-1.52	Vasospasm down vs No vasospasm
-1.36	Vasospasm down vs No vasospasm
-1.37	Vasospasm down vs No vasospasm
-1.40	Vasospasm down vs No vasospasm
-1.32	Vasospasm down vs No vasospasm
-1.23	Vasospasm down vs No vasospasm
1.39	Vasospasm up vs No vasospasm
-1.45	Vasospasm down vs No vasospasm
1.37	Vasospasm up vs No vasospasm
1.51	Vasospasm up vs No vasospasm
-1.29	Vasospasm down vs No vasospasm
-1.46	Vasospasm down vs No vasospasm
-1.33	Vasospasm down vs No vasospasm
1.50	Vasospasm up vs No vasospasm
-1.30	Vasospasm down vs No vasospasm
1.58	Vasospasm up vs No vasospasm
1.49	Vasospasm up vs No vasospasm

-1.36	Vasospasm down vs No vasospasm
-1.27	Vasospasm down vs No vasospasm
1.38	Vasospasm up vs No vasospasm
-1.38	Vasospasm down vs No vasospasm
-1.36	Vasospasm down vs No vasospasm
1.31	Vasospasm up vs No vasospasm
-1.33	Vasospasm down vs No vasospasm
-1.67	Vasospasm down vs No vasospasm
1.42	Vasospasm up vs No vasospasm
-1.45	Vasospasm down vs No vasospasm
-1.28	Vasospasm down vs No vasospasm
1.21	Vasospasm up vs No vasospasm
-1.22	Vasospasm down vs No vasospasm
-1.28	Vasospasm down vs No vasospasm
1.25	Vasospasm up vs No vasospasm
-1.43	Vasospasm down vs No vasospasm
-1.29	Vasospasm down vs No vasospasm
-1.31	Vasospasm down vs No vasospasm
-1.29	Vasospasm down vs No vasospasm
-1.54	Vasospasm down vs No vasospasm
-1.45	Vasospasm down vs No vasospasm
1.54	Vasospasm up vs No vasospasm
-1.48	Vasospasm down vs No vasospasm
-1.22	Vasospasm down vs No vasospasm
-1.22	Vasospasm down vs No vasospasm
-1.28	Vasospasm down vs No vasospasm
1.52	Vasospasm up vs No vasospasm
-1.21	Vasospasm down vs No vasospasm
1.66	Vasospasm up vs No vasospasm
-1.31	Vasospasm down vs No vasospasm
1.34	Vasospasm up vs No vasospasm
-1.38	Vasospasm down vs No vasospasm
1.53	Vasospasm up vs No vasospasm
-1.32	Vasospasm down vs No vasospasm
1.27	Vasospasm up vs No vasospasm
1.45	Vasospasm up vs No vasospasm
-1.41	Vasospasm down vs No vasospasm
1.22	Vasospasm up vs No vasospasm
1.32	Vasospasm up vs No vasospasm
-1.23	Vasospasm down vs No vasospasm
-1.30	Vasospasm down vs No vasospasm
-1.31	Vasospasm down vs No vasospasm
-1.28	Vasospasm down vs No vasospasm
-1.30	Vasospasm down vs No vasospasm
1.37	Vasospasm up vs No vasospasm
1.31	Vasospasm up vs No vasospasm
-1.21	Vasospasm down vs No vasospasm

-1.45	Vasospasm down vs No vasospasm
1.26	Vasospasm up vs No vasospasm
1.38	Vasospasm up vs No vasospasm
1.67	Vasospasm up vs No vasospasm
1.74	Vasospasm up vs No vasospasm
1.69	Vasospasm up vs No vasospasm
-1.44	Vasospasm down vs No vasospasm
-1.30	Vasospasm down vs No vasospasm
1.45	Vasospasm up vs No vasospasm
-1.32	Vasospasm down vs No vasospasm
-1.23	Vasospasm down vs No vasospasm
-1.41	Vasospasm down vs No vasospasm
1.50	Vasospasm up vs No vasospasm
-1.60	Vasospasm down vs No vasospasm
-1.37	Vasospasm down vs No vasospasm
1.64	Vasospasm up vs No vasospasm
-1.33	Vasospasm down vs No vasospasm
-1.39	Vasospasm down vs No vasospasm
1.30	Vasospasm up vs No vasospasm
-1.32	Vasospasm down vs No vasospasm
-1.31	Vasospasm down vs No vasospasm
1.42	Vasospasm up vs No vasospasm
1.30	Vasospasm up vs No vasospasm
1.59	Vasospasm up vs No vasospasm
-1.60	Vasospasm down vs No vasospasm
1.61	Vasospasm up vs No vasospasm
-1.42	Vasospasm down vs No vasospasm
-1.29	Vasospasm down vs No vasospasm
-1.27	Vasospasm down vs No vasospasm
-1.31	Vasospasm down vs No vasospasm
-1.42	Vasospasm down vs No vasospasm
1.57	Vasospasm up vs No vasospasm
-1.22	Vasospasm down vs No vasospasm
-1.54	Vasospasm down vs No vasospasm
1.95	Vasospasm up vs No vasospasm
1.72	Vasospasm up vs No vasospasm
-1.31	Vasospasm down vs No vasospasm
-1.41	Vasospasm down vs No vasospasm
1.36	Vasospasm up vs No vasospasm
1.33	Vasospasm up vs No vasospasm
1.28	Vasospasm up vs No vasospasm
1.44	Vasospasm up vs No vasospasm
-1.36	Vasospasm down vs No vasospasm
-1.21	Vasospasm down vs No vasospasm
1.63	Vasospasm up vs No vasospasm
1.28	Vasospasm up vs No vasospasm
-1.34	Vasospasm down vs No vasospasm

-1.28	Vasospasm down vs No vasospasm
1.63	Vasospasm up vs No vasospasm
-1.26	Vasospasm down vs No vasospasm
-1.21	Vasospasm down vs No vasospasm
-1.22	Vasospasm down vs No vasospasm
-1.37	Vasospasm down vs No vasospasm
1.51	Vasospasm up vs No vasospasm
1.38	Vasospasm up vs No vasospasm
1.22	Vasospasm up vs No vasospasm
1.41	Vasospasm up vs No vasospasm
1.46	Vasospasm up vs No vasospasm
1.50	Vasospasm up vs No vasospasm
-1.63	Vasospasm down vs No vasospasm
1.52	Vasospasm up vs No vasospasm
1.35	Vasospasm up vs No vasospasm
1.28	Vasospasm up vs No vasospasm
1.31	Vasospasm up vs No vasospasm
1.85	Vasospasm up vs No vasospasm
-1.31	Vasospasm down vs No vasospasm
-1.28	Vasospasm down vs No vasospasm
1.69	Vasospasm up vs No vasospasm
1.47	Vasospasm up vs No vasospasm
1.35	Vasospasm up vs No vasospasm
-1.33	Vasospasm down vs No vasospasm
-1.34	Vasospasm down vs No vasospasm
1.28	Vasospasm up vs No vasospasm
1.30	Vasospasm up vs No vasospasm
-1.54	Vasospasm down vs No vasospasm
1.48	Vasospasm up vs No vasospasm
1.85	Vasospasm up vs No vasospasm
1.36	Vasospasm up vs No vasospasm
-1.41	Vasospasm down vs No vasospasm
-1.40	Vasospasm down vs No vasospasm
-1.28	Vasospasm down vs No vasospasm
1.38	Vasospasm up vs No vasospasm
-1.39	Vasospasm down vs No vasospasm
1.61	Vasospasm up vs No vasospasm
1.45	Vasospasm up vs No vasospasm
-1.25	Vasospasm down vs No vasospasm
1.45	Vasospasm up vs No vasospasm
1.31	Vasospasm up vs No vasospasm
1.41	Vasospasm up vs No vasospasm
1.27	Vasospasm up vs No vasospasm
-1.23	Vasospasm down vs No vasospasm
-1.29	Vasospasm down vs No vasospasm
1.29	Vasospasm up vs No vasospasm
-1.29	Vasospasm down vs No vasospasm

-1.40	Vasospasm down vs No vasospasm
1.58	Vasospasm up vs No vasospasm
1.65	Vasospasm up vs No vasospasm
-1.25	Vasospasm down vs No vasospasm
-1.42	Vasospasm down vs No vasospasm
-1.23	Vasospasm down vs No vasospasm
-1.47	Vasospasm down vs No vasospasm
1.70	Vasospasm up vs No vasospasm
1.43	Vasospasm up vs No vasospasm
1.21	Vasospasm up vs No vasospasm
-1.28	Vasospasm down vs No vasospasm
-1.48	Vasospasm down vs No vasospasm
-1.42	Vasospasm down vs No vasospasm
-1.38	Vasospasm down vs No vasospasm
-1.31	Vasospasm down vs No vasospasm
1.49	Vasospasm up vs No vasospasm
-1.38	Vasospasm down vs No vasospasm
-1.50	Vasospasm down vs No vasospasm
-1.33	Vasospasm down vs No vasospasm
-1.32	Vasospasm down vs No vasospasm
-1.20	Vasospasm down vs No vasospasm
-1.71	Vasospasm down vs No vasospasm
-1.61	Vasospasm down vs No vasospasm
-1.32	Vasospasm down vs No vasospasm
-1.37	Vasospasm down vs No vasospasm
-1.35	Vasospasm down vs No vasospasm
1.56	Vasospasm up vs No vasospasm
-1.35	Vasospasm down vs No vasospasm
-1.21	Vasospasm down vs No vasospasm
1.46	Vasospasm up vs No vasospasm
1.49	Vasospasm up vs No vasospasm
-1.42	Vasospasm down vs No vasospasm
-1.27	Vasospasm down vs No vasospasm
1.22	Vasospasm up vs No vasospasm
1.27	Vasospasm up vs No vasospasm
1.25	Vasospasm up vs No vasospasm
-1.45	Vasospasm down vs No vasospasm
-1.26	Vasospasm down vs No vasospasm
-1.22	Vasospasm down vs No vasospasm
1.37	Vasospasm up vs No vasospasm
1.66	Vasospasm up vs No vasospasm
1.50	Vasospasm up vs No vasospasm
1.34	Vasospasm up vs No vasospasm
1.48	Vasospasm up vs No vasospasm
1.80	Vasospasm up vs No vasospasm
-1.27	Vasospasm down vs No vasospasm
-1.52	Vasospasm down vs No vasospasm

1.50	Vasospasm up vs No vasospasm
-1.35	Vasospasm down vs No vasospasm
-1.38	Vasospasm down vs No vasospasm
1.42	Vasospasm up vs No vasospasm
-1.26	Vasospasm down vs No vasospasm
-1.33	Vasospasm down vs No vasospasm
1.23	Vasospasm up vs No vasospasm
1.40	Vasospasm up vs No vasospasm
1.39	Vasospasm up vs No vasospasm
-1.24	Vasospasm down vs No vasospasm
-1.36	Vasospasm down vs No vasospasm
1.32	Vasospasm up vs No vasospasm
-1.41	Vasospasm down vs No vasospasm
1.87	Vasospasm up vs No vasospasm
1.36	Vasospasm up vs No vasospasm
-1.22	Vasospasm down vs No vasospasm
-1.34	Vasospasm down vs No vasospasm
-1.26	Vasospasm down vs No vasospasm
1.54	Vasospasm up vs No vasospasm
-1.23	Vasospasm down vs No vasospasm
-1.37	Vasospasm down vs No vasospasm
-1.32	Vasospasm down vs No vasospasm
1.55	Vasospasm up vs No vasospasm
-1.44	Vasospasm down vs No vasospasm
1.43	Vasospasm up vs No vasospasm
-1.32	Vasospasm down vs No vasospasm
1.56	Vasospasm up vs No vasospasm
-1.38	Vasospasm down vs No vasospasm
1.78	Vasospasm up vs No vasospasm
-1.26	Vasospasm down vs No vasospasm
-1.41	Vasospasm down vs No vasospasm
-1.23	Vasospasm down vs No vasospasm
-1.35	Vasospasm down vs No vasospasm
1.74	Vasospasm up vs No vasospasm
1.53	Vasospasm up vs No vasospasm
-1.21	Vasospasm down vs No vasospasm
-1.20	Vasospasm down vs No vasospasm
1.42	Vasospasm up vs No vasospasm
-1.37	Vasospasm down vs No vasospasm
1.35	Vasospasm up vs No vasospasm
-1.22	Vasospasm down vs No vasospasm
-1.30	Vasospasm down vs No vasospasm
-1.44	Vasospasm down vs No vasospasm
-1.30	Vasospasm down vs No vasospasm
-1.40	Vasospasm down vs No vasospasm
-1.23	Vasospasm down vs No vasospasm
-1.25	Vasospasm down vs No vasospasm

-1.37	Vasospasm down vs No vasospasm
-1.52	Vasospasm down vs No vasospasm
-1.42	Vasospasm down vs No vasospasm
-1.60	Vasospasm down vs No vasospasm
1.33	Vasospasm up vs No vasospasm
-1.36	Vasospasm down vs No vasospasm
1.65	Vasospasm up vs No vasospasm
-1.33	Vasospasm down vs No vasospasm
-1.23	Vasospasm down vs No vasospasm
1.50	Vasospasm up vs No vasospasm
-1.27	Vasospasm down vs No vasospasm
1.72	Vasospasm up vs No vasospasm
1.40	Vasospasm up vs No vasospasm
-1.49	Vasospasm down vs No vasospasm
-1.40	Vasospasm down vs No vasospasm
2.02	Vasospasm up vs No vasospasm
1.36	Vasospasm up vs No vasospasm
1.26	Vasospasm up vs No vasospasm
1.44	Vasospasm up vs No vasospasm
-1.28	Vasospasm down vs No vasospasm
1.60	Vasospasm up vs No vasospasm
-1.26	Vasospasm down vs No vasospasm
-1.29	Vasospasm down vs No vasospasm
-1.20	Vasospasm down vs No vasospasm
1.47	Vasospasm up vs No vasospasm
-1.57	Vasospasm down vs No vasospasm
1.53	Vasospasm up vs No vasospasm
-1.34	Vasospasm down vs No vasospasm
1.24	Vasospasm up vs No vasospasm
-1.42	Vasospasm down vs No vasospasm
1.60	Vasospasm up vs No vasospasm
-1.35	Vasospasm down vs No vasospasm
1.51	Vasospasm up vs No vasospasm
-1.32	Vasospasm down vs No vasospasm
1.22	Vasospasm up vs No vasospasm
-1.33	Vasospasm down vs No vasospasm
-1.38	Vasospasm down vs No vasospasm
-1.36	Vasospasm down vs No vasospasm
-1.22	Vasospasm down vs No vasospasm
1.34	Vasospasm up vs No vasospasm
1.52	Vasospasm up vs No vasospasm
-1.34	Vasospasm down vs No vasospasm
1.31	Vasospasm up vs No vasospasm
1.30	Vasospasm up vs No vasospasm
-1.30	Vasospasm down vs No vasospasm
1.48	Vasospasm up vs No vasospasm
-1.42	Vasospasm down vs No vasospasm

1.43	Vasospasm up vs No vasospasm
-1.49	Vasospasm down vs No vasospasm
-1.66	Vasospasm down vs No vasospasm
-1.32	Vasospasm down vs No vasospasm
-1.29	Vasospasm down vs No vasospasm
-1.22	Vasospasm down vs No vasospasm
-1.37	Vasospasm down vs No vasospasm
-1.46	Vasospasm down vs No vasospasm
1.47	Vasospasm up vs No vasospasm
-1.27	Vasospasm down vs No vasospasm
1.32	Vasospasm up vs No vasospasm
1.46	Vasospasm up vs No vasospasm
-1.26	Vasospasm down vs No vasospasm
-1.34	Vasospasm down vs No vasospasm
-1.20	Vasospasm down vs No vasospasm
-1.39	Vasospasm down vs No vasospasm
1.35	Vasospasm up vs No vasospasm
-1.58	Vasospasm down vs No vasospasm
-1.34	Vasospasm down vs No vasospasm
-1.32	Vasospasm down vs No vasospasm
-1.28	Vasospasm down vs No vasospasm
-1.40	Vasospasm down vs No vasospasm
-1.31	Vasospasm down vs No vasospasm
-1.38	Vasospasm down vs No vasospasm
1.40	Vasospasm up vs No vasospasm
1.36	Vasospasm up vs No vasospasm
-1.40	Vasospasm down vs No vasospasm
-1.23	Vasospasm down vs No vasospasm
1.52	Vasospasm up vs No vasospasm
1.32	Vasospasm up vs No vasospasm
-1.29	Vasospasm down vs No vasospasm
1.28	Vasospasm up vs No vasospasm
1.34	Vasospasm up vs No vasospasm
-1.21	Vasospasm down vs No vasospasm
1.98	Vasospasm up vs No vasospasm
1.27	Vasospasm up vs No vasospasm
-1.26	Vasospasm down vs No vasospasm
-1.23	Vasospasm down vs No vasospasm
1.33	Vasospasm up vs No vasospasm
-1.26	Vasospasm down vs No vasospasm
-1.48	Vasospasm down vs No vasospasm
-1.31	Vasospasm down vs No vasospasm
1.66	Vasospasm up vs No vasospasm
1.23	Vasospasm up vs No vasospasm
-1.27	Vasospasm down vs No vasospasm
1.42	Vasospasm up vs No vasospasm
1.26	Vasospasm up vs No vasospasm

-1.35	Vasospasm down vs No vasospasm
1.43	Vasospasm up vs No vasospasm
1.78	Vasospasm up vs No vasospasm
-1.35	Vasospasm down vs No vasospasm
-1.26	Vasospasm down vs No vasospasm
-1.22	Vasospasm down vs No vasospasm
-1.53	Vasospasm down vs No vasospasm
-1.29	Vasospasm down vs No vasospasm
1.58	Vasospasm up vs No vasospasm
-1.33	Vasospasm down vs No vasospasm
-1.32	Vasospasm down vs No vasospasm
1.44	Vasospasm up vs No vasospasm
1.31	Vasospasm up vs No vasospasm
-1.51	Vasospasm down vs No vasospasm
1.41	Vasospasm up vs No vasospasm
1.44	Vasospasm up vs No vasospasm
-1.38	Vasospasm down vs No vasospasm
-1.28	Vasospasm down vs No vasospasm
1.37	Vasospasm up vs No vasospasm
1.48	Vasospasm up vs No vasospasm
-1.30	Vasospasm down vs No vasospasm
1.28	Vasospasm up vs No vasospasm
1.32	Vasospasm up vs No vasospasm
-1.29	Vasospasm down vs No vasospasm
-1.33	Vasospasm down vs No vasospasm
-1.50	Vasospasm down vs No vasospasm
-1.42	Vasospasm down vs No vasospasm
-1.30	Vasospasm down vs No vasospasm
1.52	Vasospasm up vs No vasospasm
-1.21	Vasospasm down vs No vasospasm
-1.28	Vasospasm down vs No vasospasm
-1.43	Vasospasm down vs No vasospasm
1.60	Vasospasm up vs No vasospasm
-1.27	Vasospasm down vs No vasospasm
-1.26	Vasospasm down vs No vasospasm
1.51	Vasospasm up vs No vasospasm
1.40	Vasospasm up vs No vasospasm
-1.25	Vasospasm down vs No vasospasm
-1.29	Vasospasm down vs No vasospasm
1.47	Vasospasm up vs No vasospasm
-1.25	Vasospasm down vs No vasospasm
1.36	Vasospasm up vs No vasospasm
-1.29	Vasospasm down vs No vasospasm
1.75	Vasospasm up vs No vasospasm
-1.21	Vasospasm down vs No vasospasm
-1.40	Vasospasm down vs No vasospasm
-1.23	Vasospasm down vs No vasospasm

1.43	Vasospasm up vs No vasospasm
-1.30	Vasospasm down vs No vasospasm
-1.32	Vasospasm down vs No vasospasm
-1.62	Vasospasm down vs No vasospasm
1.26	Vasospasm up vs No vasospasm
1.44	Vasospasm up vs No vasospasm
-1.75	Vasospasm down vs No vasospasm
-1.52	Vasospasm down vs No vasospasm
1.38	Vasospasm up vs No vasospasm
-1.41	Vasospasm down vs No vasospasm
1.36	Vasospasm up vs No vasospasm
1.54	Vasospasm up vs No vasospasm
1.31	Vasospasm up vs No vasospasm
-1.28	Vasospasm down vs No vasospasm
1.36	Vasospasm up vs No vasospasm
1.65	Vasospasm up vs No vasospasm
-1.29	Vasospasm down vs No vasospasm
1.30	Vasospasm up vs No vasospasm
1.20	Vasospasm up vs No vasospasm
1.45	Vasospasm up vs No vasospasm
-1.24	Vasospasm down vs No vasospasm
-1.27	Vasospasm down vs No vasospasm
1.54	Vasospasm up vs No vasospasm
-1.51	Vasospasm down vs No vasospasm
2.12	Vasospasm up vs No vasospasm
-1.25	Vasospasm down vs No vasospasm
1.47	Vasospasm up vs No vasospasm
1.20	Vasospasm up vs No vasospasm
-1.52	Vasospasm down vs No vasospasm
-1.47	Vasospasm down vs No vasospasm
-1.46	Vasospasm down vs No vasospasm
-1.52	Vasospasm down vs No vasospasm
1.31	Vasospasm up vs No vasospasm
-1.30	Vasospasm down vs No vasospasm
1.77	Vasospasm up vs No vasospasm
-1.41	Vasospasm down vs No vasospasm
